# Lack of myotubularin phosphatase activity is the main cause of X-linked myotubular myopathy

**DOI:** 10.1172/jci.insight.189286

**Published:** 2025-10-14

**Authors:** Foteini Moschovaki-Filippidou, Christine Kretz, David Reiss, Gaëtan Chicanne, Bernard Payrastre, Jocelyn Laporte

**Affiliations:** 1 IGBMC (Institut de Génétique et de Biologie Moléculaire et Cellulaire), Inserm UMR-S 1258, CNRS UMR7104, Université de Strasbourg, F67404 Illkirch, France.; 2 I2MC (Institut des Maladies Métaboliques et Cardiovasculaires), Inserm UMR 1297, University of Toulouse, Toulouse, France.

**Keywords:** Genetics, Muscle biology, Mitochondria, Mouse models, Skeletal muscle

## Abstract

The *MTM1* gene encodes myotubularin (MTM1), a phosphatidylinositol 3-phosphate [PI(3)P] lipid phosphatase. Loss-of-function mutations in *MTM1* cause X-linked myotubular myopathy (XLMTM), a severe congenital myopathy with no available cure and a poorly understood pathomechanism. The importance of MTM1 enzymatic activity and its PI(3)P substrate in physiology under normal conditions and in XLMTM is unclear. We generated the *Mtm1-*KI C375S mice in which the endogenous MTM1 was converted to a phosphatase-dead protein. Mutant mice survived a median of 12 weeks and demonstrated progressively impaired motor skills. Observed muscle hypotrophy and reduced force production compared with their WT littermates (~3.9-fold reduction in absolute maximal force) were responsible for these severe phenotypes. A significantly higher level of PI(3)P was found in the muscle of *Mtm1-*KI C375S mice. Muscle histology and molecular characterization revealed XLMTM hallmarks, with (a) alteration of the mTOR and autophagy pathways correlating with muscle hypotrophy and (b) abnormal myofiber intracellular organization correlating with impaired muscle force. Overall, this study reveals the importance of MTM1 phosphatase activity and related PI(3)P substrate for postnatal muscle maintenance, and it highlights the significance of MTM1 phosphatase activity in the development of X-linked myotubular myopathy.

## Abstract 2

Mice with phosphatase-dead myotubularin develop severe myopathy, emphasizing the critical importance of the protein’s enzymatic activity in sustaining healthy muscle structure and function.

## Graphical abstract

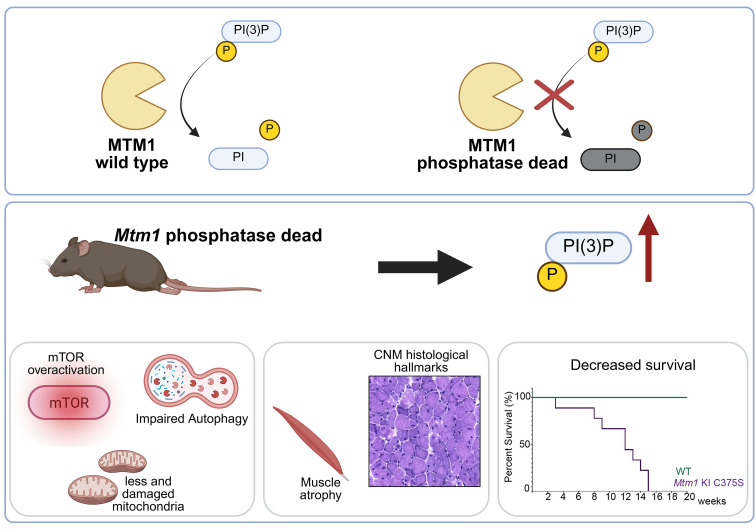

## Introduction

X-linked myotubular myopathy (XLMTM), also called X-linked centronuclear myopathy, is a severe congenital muscle disorder that predominantly affects males and occurs in approximately 1 in 50,000 births worldwide (1, 2). Clinically, XLMTM is characterized by significant muscle weakness, muscle hypotrophy, and respiratory distress and most often results in death within the first months or years of life (3–5). Histological hallmarks include smaller muscle fibers with centralized nuclei (6). There is currently no cure for XLMTM, and its pathomechanism remains poorly understood.

Mutations in the *MTM1* gene are responsible for XLMTM. These mutations occur throughout the *MTM1* gene and are recognized as loss-of-function mutations (7–11). Indeed, the vast majority correlates with a strong decrease or absence of detection of the MTM1 protein (12).

The *MTM1* gene codes for the protein myotubularin (MTM1) that defined a large family of proteins conserved through evolution, with several paralogs mutated in peripheral neuropathies (13–16). MTM1 contains protein-protein and protein-lipid binding domains as the PH-GRAM, Rac-induced recruitment domain (RID), SET-protein interaction domain (SID), and coiled-coil and PDZ-binding domains, and it also contains a phosphoinositides phosphatase catalytic domain (7, 14, 17–22). Mutation of the catalytic cysteine to serine (C375S) results in a complete loss of its enzymatic activity (23, 24). Under normal physiological conditions, MTM1 dephosphorylates phosphatidylinositol 3-phosphate [PI(3)P] to phosphatidylinositol (PI) and phosphatidylinositol 3,5-bisphosphate [PI(3,5)P_2_] to phosphatidylinositol 5-phosphate [PI(5)P], contributing to the maintenance of phosphoinositides balance within cells (23–25). Through its different protein domains, MTM1 plays a crucial role in membrane trafficking and remodeling and in excitation-contraction coupling (26–29).

Several animal models lacking the MTM1 protein were previously generated and characterized. In mice, 3 different mouse lines were generated through either a KO approach (*Mtm1*^–/y^) (30), a gene trap (*Mtm1*^gt/y^) (31), or a knock-in of a patient mutation (*Mtm1* p.R69C) (32), all leading to the loss of MTM1. As the first mouse model created at IGBMC (Illkirch, France), the *Mtm1*^–/y^ mouse is a faithful model for XLMTM, accurately reproducing the muscle weakness and histology patterns observed in patients. These mice typically survive approximately until 7 weeks of age. Already at 3 weeks, mice develop a progressive and generalized myopathy with lower body mass (30, 33).

Multi-omics analysis at different time points and in different genetic backgrounds indicates defects in muscle contraction, sarcomere organization, and cell adhesion as potential disease causes and highlights the significance of the abnormalities in neuromuscular junction and the dysregulations in basement membrane pathways (33, 34). Additionally, a recent study has elegantly highlighted the crucial role of MTM1 in maintaining mitochondrial function by coordinating the ER and mitochondria dynamics through PI(3)P regulation on endosomes (35). Finally, overactivation of mTOR and the effect on the LC3 and p62 protein levels in the muscles of *Mtm1*^–/y^ mice has led to the hypothesis of the mutation suppressing autophagy (31, 36). Dynamin 2 (DNM2) and amphiphysin 2 (BIN1) are also proteins involved in membrane dynamics, are mutated in autosomal forms of centronuclear myopathy and have been implicated in the same molecular pathway together with MTM1 (37–40). In the muscles of *Mtm1*^–/y^ mice, both DNM2 and BIN1 protein levels are elevated while downregulation of DNM2 and further upregulation of BIN1 rescues *Mtm1*^–/y^ mice from the myopathic phenotypes (41, 42).

Overall, the importance of the phosphatase activity of MTM1 in muscle physiology and in the XLMTM pathology is still unclear. In particular, apparent discrepancies have been reported. While the *Mtm1*^–/y^ mouse can be efficiently rescued by gene replacement with exogenous WT MTM1, most of the phenotypes are also rescued with the MTM1-C375S phosphatase-dead or a second phosphatase inactive mutant MTM1-S376N (43). Conversely, *Mtm1*^–/y^ mice can also be rescued by decreasing the production of PI(3)P through genetic crosses with mice either lacking the class II PI 3-kinase PIK3C2B (44) or specifically lacking the enzymatic activity of PIK3C2B (45), or through treatment with wortmannin, a broad inhibitor of PI 3-kinases (46)

Here, we aimed to resolve this controversy and investigate the importance of MTM1 phosphatase activity in XLMTM. For this purpose, we developed a knock-in mouse model that ubiquitously expresses phosphatase-dead MTM1 (C375S) in place of WT MTM1. We performed a full characterization of this mouse model through behavioral, motor, physiological, histological, ultrastructural, and molecular assessments and deciphered the molecular pathways specifically linked to the regulation of phosphoinositides level by MTM1.

## Results

### Generation and validation of a mouse model lacking MTM1 phosphatase activity.

**Figure 1:**
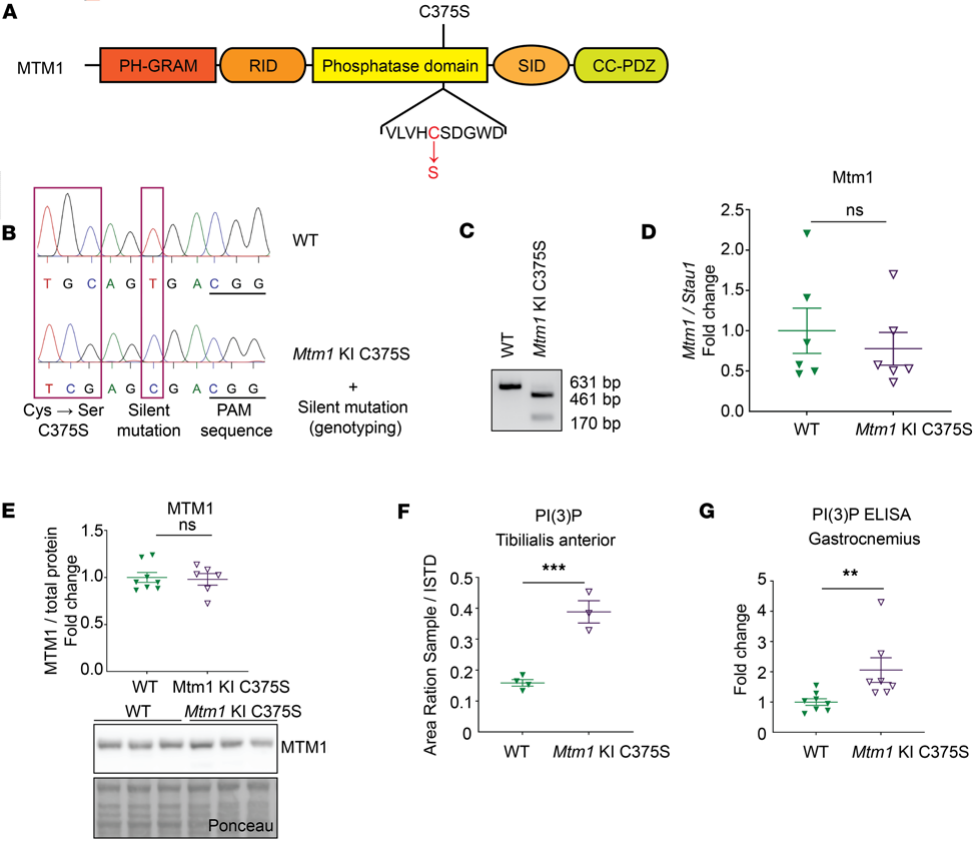
Generation and validation of the *Mtm1*-KI C375S mouse. (**A**) Representation of the MTM1 protein with its domains, the position of the C375S mutation, and the amino acid sequence around this position. Highlighted in red the catalytic cysteine, replaced by a serine in the mutant. (**B**) Chromatograms of WT and *Mtm1*-KI C375S mice. The red boxed indicate the mutation sites and the red arrows the silent mutation introduced for genotyping purposes. (**C**) Example of genotyping after PCR products were digested with the XhoI enzyme. WT band at 631 bp and *Mtm1*-KI C375S band at 461 and 170 bp. (**D**) *Mtm1* mRNA levels in the tibialis anterior (TA) muscle of 8-week-old WT and *Mtm1*-KI C375S mice (*n* = 6). (**E**) MTM1 protein levels in the TA muscle of 8-week-old WT and *Mtm1*-KI C375S mice. WT, *n* = 8; *Mtm1*-KI C375S, *n* = 6. (**F**) PI(3)P levels in the TA muscle of *Mtm1*-KI C375S mice and their WT littermates at 8 weeks of age as measured by mass spectrometry. WT, *n* = 4; *Mtm1*-KI C375S, *n* = 3. (**G**) PI(3)P levels measured by a commercial ELISA kit in the gastrocnemius muscle of 8-week-old WT and *Mtm1*-KI C375S mice. WT, *n* = 8; *Mtm1*-KI C375S, *n* = 7. Mann-Whitney 2-tailed test for MTM1 protein and PI(3)P levels measured by ELISA; unpaired 2-tailed *t* test for *Mtm1* mRNA levels and PI(3)P levels measured by mass spectrometry; **P ≤ 0.01, ***P ≤ 0.001. Data are shown as mean ± SEM.

We aimed to develop a novel mouse model expressing MTM1 that lacks phosphatase activity. Since the *Mtm1* gene is located on the X chromosome, all our experiments were conducted on male mice. To create the model, we introduced the C375S mutation in the phosphatase domain by replacing the catalytic cysteine with a serine (Figure 1A). Additionally, a silent point mutation was included to facilitate genotyping of the mice by creating a XhoI restriction site (Figure 1, B and C). The presence of these mutations was confirmed by Sanger sequencing (Figure 1B). To validate this model, we firstly demonstrated that MTM1 protein and mRNA levels in muscles were not affected by the mutation and were similar between WT and *Mtm1*–knock-in C375S mice at 8 weeks of age (Figure 1, D and E). Additionally, MTM1 protein levels were evaluated at a later age of 12 weeks, confirming the presence and stability of the C375S mutant protein over time (Supplemental Figure 1A; supplemental material available online with this article; https://doi.org/10.1172/jci.insight.189286DS1). The subcellular localization of the mutant protein appeared broadly similar to that of the WT, with no major detectable changes based on our qualitative assessment (Supplemental Figure 1B). Nevertheless, the PI(3)P levels were significantly higher in the muscles of *Mtm1*-KI C375S mice compared with WT mice (almost 2.5-fold increase in the tibialis anterior (TA) and 2-fold increase in the gastrocnemius), confirming the catalytic inactivity of MTM1 in mutant mice, although contributions from other cell types within the muscle cannot be fully excluded (Figure 1, F and G). No differences were observed in the *PI3KC2b* mRNA levels in the studied muscles, while there was a significant downregulation in the *Mtmr1*, *Mtmr2* and *Mtmr5* but not *Mtmr13* mRNA levels in the TA muscles of *Mtm1*-KI C375S mice (Supplemental Figure 8). These data confirm the *Mtm1*-KI C375S mouse expresses a normal level of phosphatase-dead MTM1.

### Mtm1-KI C375S mice present a decreased survival and a progressive myopathy.

**Figure 2:**
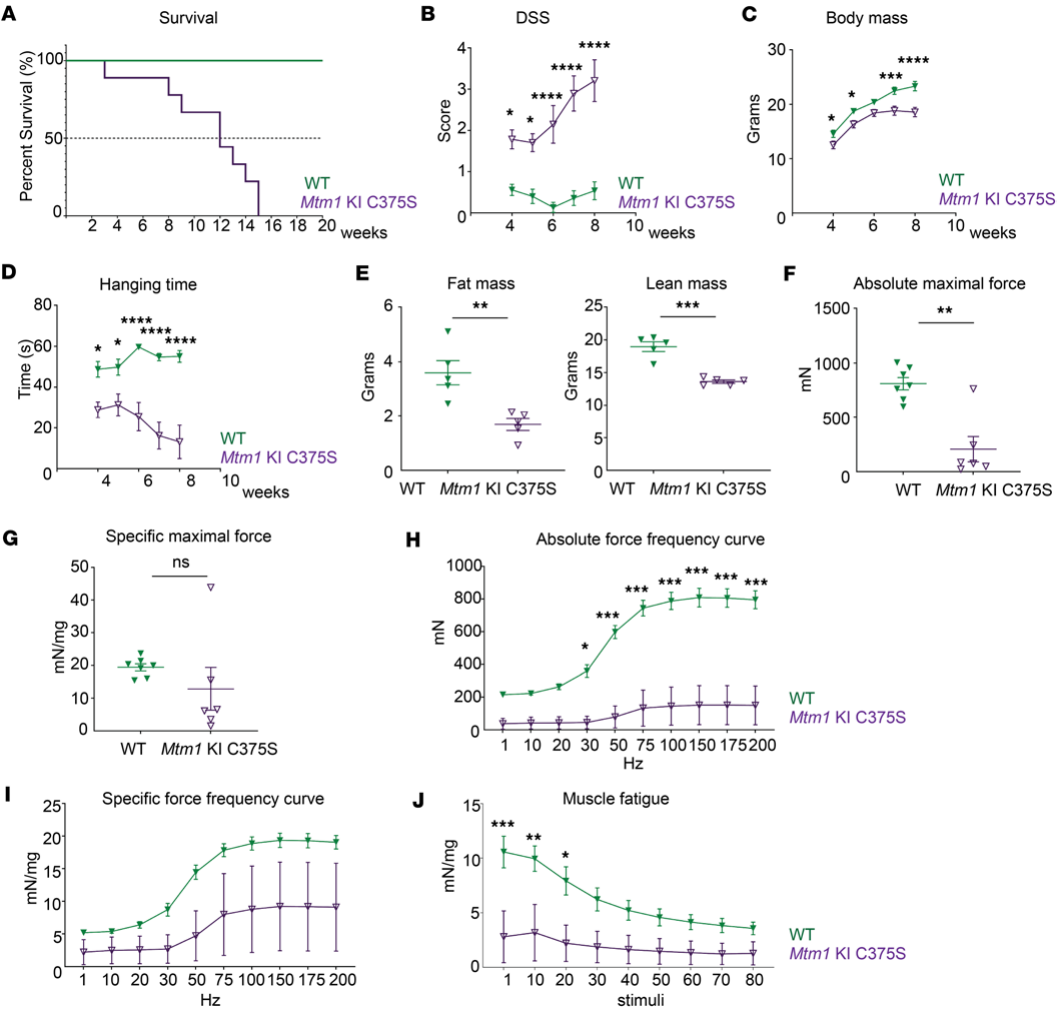
Decreased survival and progressive muscle weakness in the *Mtm1*-KI C375S mouse model. (**A**) Percentage of survival of *Mtm1*-KI C375S (*n* = 9) and WT mice (*n* = 3). (**B**–**D**) Disease severity score, total body mass, and performance on hanging test over 4 weeks of phenotyping, performed weekly, for WT and *Mtm1*-KI C375S mice. WT, *n* = 8; *Mtm1*-KI C375S, *n* = 7. (**E**) Fat mass and lean mass of WT and *Mtm1*-KI C375S mice at 8 weeks of age (*n* = 5). (**F** and **G**) Absolute and specific maximal force measured in situ in the TA muscle stimulated at 150 Hz, in *Mtm1*-KI C375S mice and their WT littermates at 8 weeks of age. WT, *n* = 7; *Mtm1*-KI C375S, *n* = 6. (**H**) Absolute TA force measured in situ following different frequency stimulations in 8-week-old *Mtm1*-KI C375S and WT mice. WT, *n* = 7; *Mtm1*-KI C375S, *n* = 6. (**I**) Specific submaximal and maximal force of the TA muscle from in situ measurements following stimulations at different frequencies, in 8-week-old *Mtm1*-KI C375S mice and their WT littermates. WT, *n* = 7; *Mtm1*-KI C375S, *n* = 6. (**J**) Specific force production by the TA muscle in situ during 80 stimulations at 40Hz, in *Mtm 1*-KI C375S mice and their WT littermates at 8 weeks of age. WT, *n* = 7; *Mtm1*-KI C375S, *n* = 6. Ordinary 2-way ANOVA with Šídák multiple comparisons for DSS, body mass, hanging test, force-frequency curves and fatigue test; Mann-Whitney 2-tailed test for absolute maximal force; unpaired 2-tailed *t* test with Welch’s corrections for fat mass, lean mass and specific maximal force; *P ≤ 0.05, **P ≤ 0.01, ***P ≤ 0.001, ****P ≤ 0.0001. Data are shown as mean ± SEM.

Having validated the expression of phosphatase-dead MTM1 by *Mtm1*-KI C375S mice, we investigated the effect of the specific loss of the phosphatase activity while nonenzymatic functions of MTM1 were conserved. *Mtm1*-KI C375S mice survived a median of 12 weeks while the longest survivor reached 15 weeks of age (Figure 2A). Though the lifespan of mutant mice was significantly reduced compared with that of WT animals, it should be noted that it was longer than what has been reported for *Mtm1*^–/y^ mice, which — depending on the genetic background — survive a median of approximately 5–7 weeks (30, 33, 38, 41, 47–51). For assessing the disease progression, we monitored the mice from 4 to 8 weeks of age and evaluated their motor abilities and general clinical condition at weekly intervals. By the age of 4 weeks, the mice exhibited a high disease severity score, indicating motor disabilities, reduced body mass, and the presence of spinal curvature, further supporting that the *Mtm1*-KI C375S mice were affected by the lack of phosphatase activity. Over the next 4 weeks, their scores increased, pointing to worsening health and disease progression (Figure 2B and Supplemental Video). When 4 weeks old, *Mtm1*-KI C375S mice showed a 1.16-fold decrease in their total body mass compared with their WT littermates. By 8 weeks of age, this difference increased to 1.26-fold, with the *Mtm1*-KI C375S mice reaching an average total body mass of 18.6 grams (Figure 2C). Notably, *Mtm1*^–/y^ mice are well documented to plateau at around 15 gr (30, 33, 38, 47, 48). When 8 weeks old, the mice underwent a total body composition analysis by Nuclear Magnetic Resonance that revealed lower fat and lean mass in comparison to their WT littermates (Figure 2E). These data indicate that a generally altered body composition is a major contributing factor to the lower body mass. Throughout the 4-week study period, *Mtm1*-KI C375S mice consistently underperformed on the hanging test in comparison with their WT littermates, indicating compromised motor function. A noticeable drop in their ability to hang was observed starting at 6 weeks of age, finally leading to a 2.2-fold decrease in the hanging time compared with their performance when 4 weeks old (Figure 2D). These data, supporting their impaired motor abilities especially at the later time points of the study, aligned with the results from the in situ muscle force measurements on the TA muscle, performed at 8-week-old mice. The absolute maximal force production was almost 4 times lower in the *Mtm1*-KI C375S mice compared with WT animals. The specific maximal force produced by the *Mtm1*-KI C375S mice was notably lower at an average of 12.8 mN/mg compared with 19.4 mN/mg for the WT mice, though this difference did not reach statistical significance due to high variation within the mutant group. The mutation’s less profound effect on specific force production compared with absolute force production indicates that the force defects in the model primarily result, at least in part, from muscle hypotrophy (Figure 2, F and G). The absolute force frequency curve revealed that mutant muscles have a strong weakness at all stimulation frequencies while normalization on the muscle mass partly reduce the difference (Figure 2, H and I). It should be noted that the specific force produced by the TA muscle in *Mtm1*-KI C375S mice was substantially higher than previously reported values in *Mtm1*^–/y^ mice, where, for example, the maximal specific force was recorded at just around 5 mN/mg (38, 41). Finally, to investigate the muscle fatigability, we performed multiple contraction at 40 Hz. The *Mtm1*-KI C375S do not show a significant drop in the produced force, as — already at the first stimulations — they fail to develop significant force (Figure 2J). Blood and plasma analysis from samples collected at 8 weeks of age showed no differences in the liver enzyme levels and the blood cell counts between *Mtm1*-KI C375S and WT mice (Supplemental Figures 2 and 3). Taken together, these results support that mice expressing the MTM1 protein orphaned of its phosphatase activity have a shortened lifespan and develop progressive muscle weakness, similarly to mice completely lacking the protein but possibly with a lower severity.

### Muscle hypotrophy and severe structural disorganization in muscles of Mtm1-KI C375S mice.

**Figure 3:**
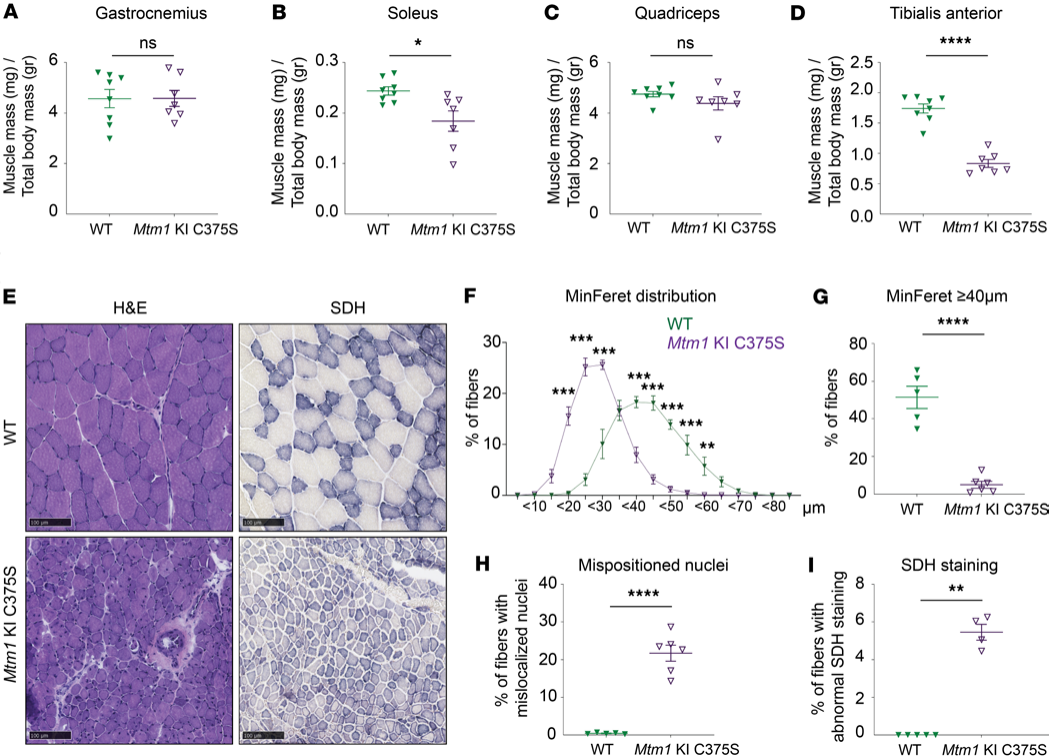
Muscle and myofibers hypotrophy and histological XLMTM hallmarks in *Mtm1*-KI C375S mouse model. (**A**–**D**) Normalized gastrocnemius, soleus, quadriceps, and TA mass in *Mtm1*-KI C375S mice and their WT littermates at 8 weeks of age. WT, *n* = 8; *Mtm1*-KI C375S, *n* = 7. (**E**) Representative pictures of TA muscle sections stained with H&E or SDH from 8-week-old WT and *Mtm1*-KI C375S mice. Scale bar: 100 μm. (**F**) MinFeret diameter distribution of TA fibers in *Mtm1*-KI C375S mice and their WT littermates at 8 weeks of age. WT, *n* = 5; *Mtm1*-KI C375S, *n* = 6. (**G**) Percentage of large fibers with MinFeret diameter ≥ 40 μm. WT, *n* = 5; *Mtm1*-KI C375S, *n* = 6. (**H**) Percentage of fibers with mispositioned nuclei in TA muscle sections from 8-week-old WT and *Mtm1*-KI C375S mice. WT, *n* = 5; *Mtm1*-KI C375S, *n* = 6. (**I**) Percentage of fibers with abnormal SDH staining in TA muscle sections from 8-week-old WT and *Mtm1*-KI C375S mice. WT, *n* = 5; *Mtm1*-KI C375S, *n* = 4. Unpaired 2-tailed *t* test for gastrocnemius, quadriceps, and TA mass; unpaired 2-tailed *t* test with Welch’s corrections for soleus mass; ordinary 2-way ANOVA with Šídák multiple comparisons for MinFeret distribution curves; *P ≤ 0.05, **P ≤ 0.01, ***P ≤ 0.001, ****P ≤ 0.0001. Data are shown as mean ± SEM.

The *Mtm1*-KI C375S mice present with reduced muscle mass and force, as for patients with XLMTM. We investigated the underlying reason through the characterization of different muscles and of myofibers. No hypotrophy was noted for the gastrocnemius or the quadriceps muscles in *Mtm1*-KI C375S mice compared with WT controls (Figure 3, A and C). This finding is noteworthy, as the gastrocnemius has previously been reported to exhibit a significantly reduced mass in the *Mtm1*^–/y^ mouse model when compared with WT animals, even at the early timepoint of 5 weeks (33, 38, 41). The soleus muscles of the *Mtm1*-KI C375S mice had a significantly smaller mass than that of WT mice, and the normalized weight of the TA muscle in mutant mice showed a 2.1-fold reduction compared with that of their WT littermates (Figure 3, B and D). The absolute muscle mass values are given in Supplemental Figure 4, A–D. As TA was the most affected muscle, we then focused on this muscle for histological analyses.

The histological analysis of the TA muscle revealed significant myofiber hypotrophy for the *Mtm1*-KI C375S mice, with a switch toward smaller fibers and a percentage of large fibers with MinFeret diameter above 40 μm 10 times lower than for the WT mice (Figure 3, E–G, and Supplemental Figure 4E). Furthermore, we found more than 20% of the total fibers analyzed to have mispositioned nuclei, a characteristic XLMTM hallmark (Figure 3, E and H). In the gastrocnemius muscle, histological analysis similarly revealed typical XLMTM hallmarks, although these defects were less pronounced and reached lower statistical significance in all analyzed parameters compared with the TA muscle (Supplemental Figure 5, A–E). These observations appear to correlate with the higher relative increase in PI(3)P levels observed in the TA muscle of mutant mice compared with their WT littermates, which appears more evident than the increase detected in the gastrocnemius (Figure 1, F and G).

**Figure 4:**
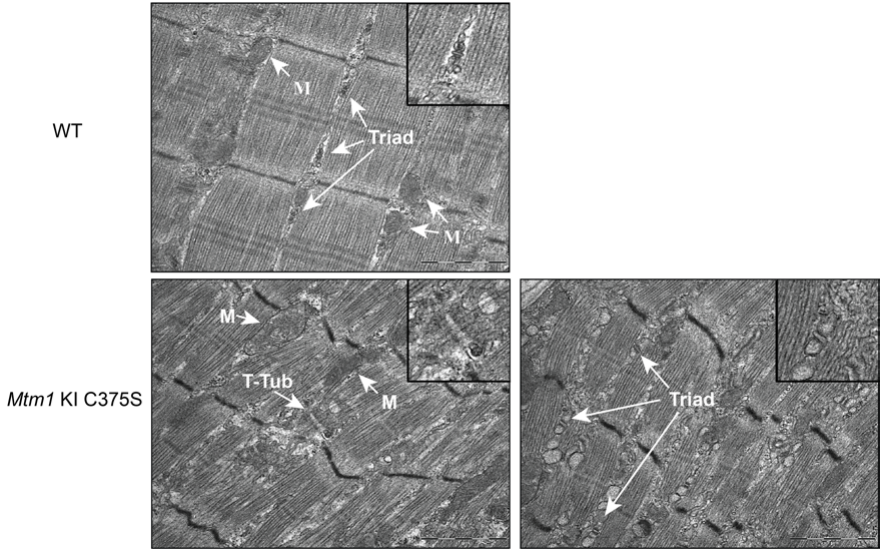
Severe structural disorganization in myofibers of *Mtm1*-KI C375S mice. Representative TA muscle pictures by electron microscopy from 8-week-old WT and *Mtm1*-KI C375S mice. Mitochondria (M), t-tubules (T-Tub), and triads are indicated in the picture. Scale bar: 1 μm.

**Figure 5:**
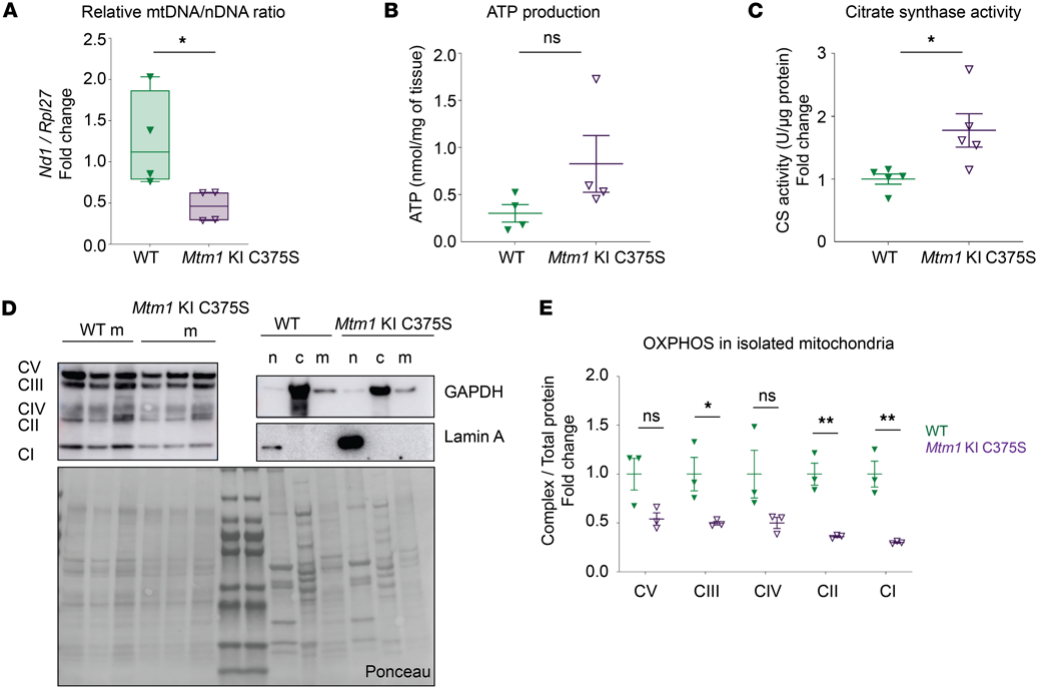
Mitochondrial defects in the TA muscles of *Mtm1*-KI C375S mice. The TA muscles of 8-week-old *Mtm1*-KI C375S mice and their age-matched WT littermates were analyzed. (**A**) Mitochondrial DNA to nuclear DNA ratio (*n* = 4). (**B** and **C**) ATP production and citrate synthase (CS) activity as measured by commercial kits (*n* = 4). (**D**) Immunoblots demonstrating successful subcellular fractionation based on the relative expression of OXPHOS complexes, GAPDH, and Lamin A. n, nuclear fraction; c, cytoplasmic fraction; m, mitochondrial fraction. Mix of 3 mice per sample for GAPDH and Lamin A. (**E**) OXPHOS complexes levels in the isolated mitochondrial subcellular fraction (*n* = 3). Mann-Whitney 2-tailed test for mitochondrial/nuclear DNA ratio, ATP production and OXPHOS CV complex protein levels; unpaired 2-tailed *t* test for all other comparisons; *P ≤ 0.05, **P ≤ 0.01. Data are shown as mean ± SEM.

Abnormal succinate dehydrogenase (SDH) staining was discovered in more than 5% of the studied fibers, indicating accumulation of oxidative activity, with central or most often subsarcolemmal excessive mislocalization of mitochondria (Figure 3, E and I). Consistent with this result, structural mitochondrial defects and abnormally sized mitochondria were prevalently observed in electron microscopy images of the TA muscles of *Mtm1*-KI C375S mice (Figure 4). Moreover, the relative mitochondrial/nuclear DNA ratio was 2 times lower in the TA muscles of *Mtm1*-KI C375S mice compared with their WT littermates (Figure 5A). Nevertheless, ATP production was not significantly affected, while citrate synthase activity was higher in *Mtm1*-KI C375S mouse TA muscles, indicating an underlying compensatory mechanism at play (Figure 5, B and C). Western blot analysis on the isolated mitochondrial fraction of these muscles showed dysregulated protein levels for complexes of the mitochondrial oxidative phosphorylation system (OXPHOS), additionally indicating damaged mitochondria (Figure 5, D and E). Finally, a 1.3-fold decrease was found for prohibitin protein levels, a marker of the inner mitochondrial membrane (Supplemental Figure 7). No changes were noted in the percentage of type IIa, IIb, or IIx/d within genotypes in the TA muscles, but a significant increase in type I fibers in the TA of mutant animals (Supplemental Figure 6).

Further analysis of the muscle ultrastructure by electron microscopy revealed severely disorganized and smaller sarcomeres with misaligned Z lines and notably lacking normal triad structures (Figure 4). Overall, these findings support that the potentially novel *Mtm1*-KI C375S mouse model faithfully exhibits all the XLMTM hallmark features in histology, muscle ultrastructure, and mitochondrial defects.

### The phosphatase activity of MTM1 is required for normal muscle DNM2 and BIN1 protein levels.

**Figure 6:**
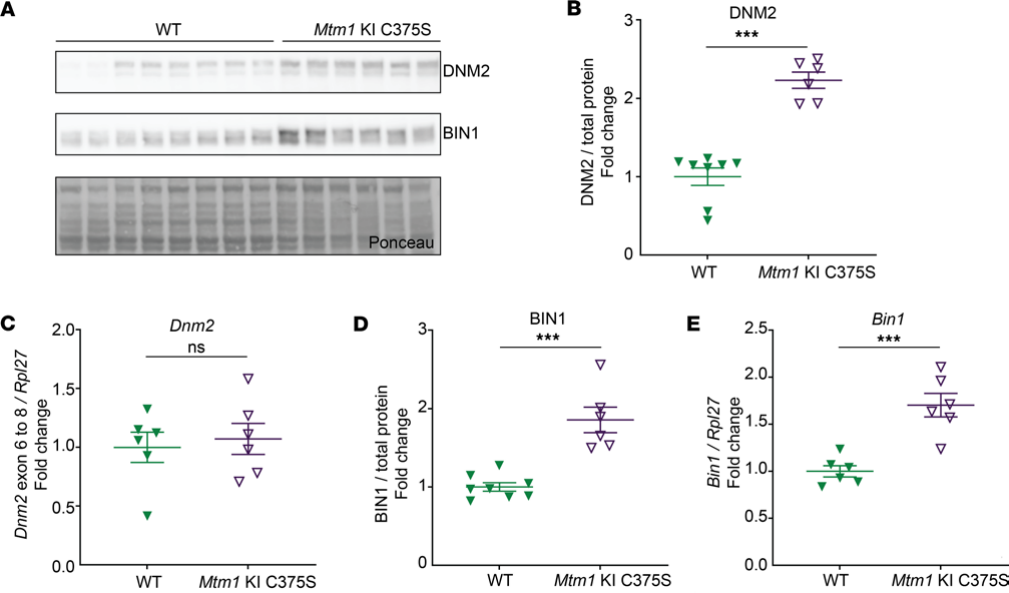
DNM2 and BIN1 protein levels are affected in the muscles of *Mtm1*-KI C375S mice. (**A**) Representative blots for DNM2 and BIN1 protein levels in the TA muscles of WT mice and their *Mtm1*-KI C375S littermates at 8 weeks of age. (**B** and **C**) DNM2 protein levels and *Dnm2* mRNA levels in the TA muscles of 8-week-old WT and *Mtm1*-KI C375S mice. For protein WT, *n* = 8; *Mtm1*-KI C375S, *n* = 6. For mRNA, *n* = 6. (**D** and **E**) BIN1 protein levels and *Bin1* mRNA levels in the TA muscles of 8-week-old WT and *Mtm1*-KI C375S mice. For protein WT, *n* = 8; *Mtm1*-KI C375S, *n* = 6. For mRNA, *n* = 6. Mann-Whitney 2-tailed test for DNM2 protein levels; unpaired 2-tailed *t* test for all other comparisons; ***P ≤ 0.001. Data are shown as mean ± SEM.

Considering the significance of DNM2 protein levels in the XLMTM pathology and the role of BIN1 as a negative regulator of DNM2 (39), we next investigated this pathway in the muscles of *Mtm1*-KI C375S mice. Similar to observations in the *Mtm1*^–/y^ mice and in patients with XLMTM (38), we noted significantly higher DNM2 protein levels in the TA muscles of *Mtm1*-KI C375S mice compared with WT mice (2.2-fold), while the *Dnm2* mRNA levels remained unchanged (Figure 6, A–C). BIN1 protein and mRNA levels were 1.9-fold and 1.7-fold higher, respectively, in the muscles of the mutant mice (Figure 6, A, D and E). This is also consistent with findings in *Mtm1*^–/y^ mice (39). Similar results were observed in the gastrocnemius muscles of *Mtm1*-KI C375S mice (Supplemental Figure 5, F and G). Taken together, it supports the idea that DNM2 plays a causative role in the disease progression, while changes in BIN1 levels are consequential and act as a compensatory mechanism through transcription adaptation. In conclusion, these data show that DNM2 protein level is regulated by the enzymatic activity of MTM1.

### Overactivation of mTOR in muscles of Mtm1-KI C375S mice.

**Figure 7:**
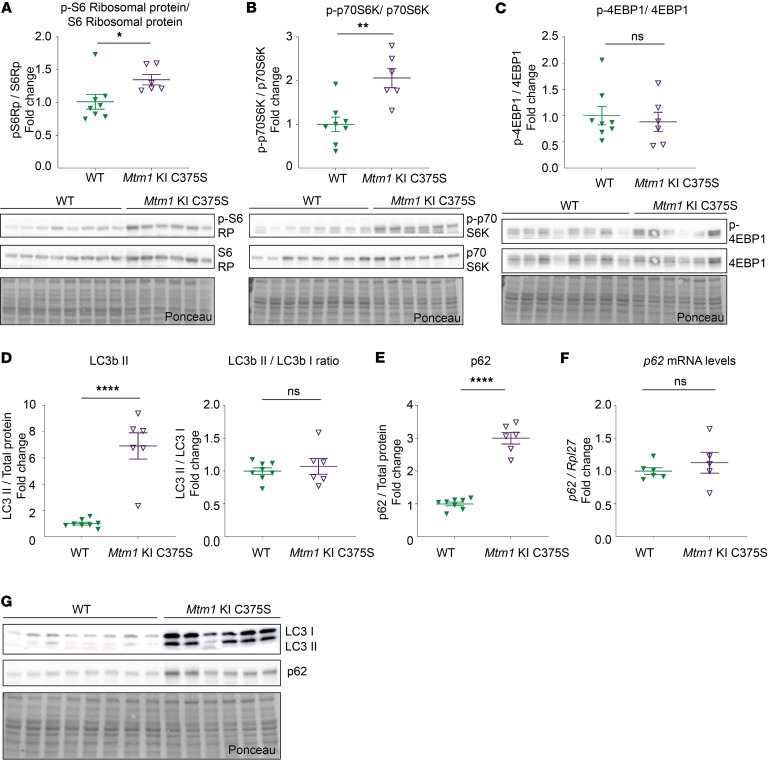
mTOR overactivation and autophagy alteration in the muscles of *Mtm1*-KI C375S mice. (**A**–**C**) Protein levels of phosphorylated and total S6 Ribosomal protein phosphorylated and total 70S6K and phosphorylated and total 4EBP1 in the TA muscles of 8-week-old WT and *Mtm1*-KI C375S mice. WT, *n* = 8; *Mtm1*-KI C375S, *n* = 6. (**D**) LC3b II protein levels and the LC3b II/LC3b I ratio in the TA muscle of WT and *Mtm1*-KI C375S mice at 8 weeks of age. WT, *n* = 8; *Mtm1*-KI C375S, *n* = 6. (**E** and **F**) p62 protein levels (WT, *n* = 8; *Mtm1*-KI C375S, *n* = 6) and mRNA levels (WT, *n* = 6; *Mtm1*-KI C375S, *n* = 5) in the TA muscles of *Mtm1*-KI C375S mice and their WT littermates. (**G**) Western blot images for LC3b I, LC3b II, p62, and total protein. Mann-Whitney 2-tailed test for S6 Ribosomal protein phosphorylation and 4EBP1 phosphorylation; unpaired 2-tailed *t* test for all other comparisons; *P ≤ 0.05, **P ≤ 0.01, ****P ≤ 0.0001. Data are shown as mean ± SEM.

MTM1 homologs and PI(3)P were previously implicated in the regulation of the mTOR pathway (52). Moreover, previous studies have reported mTOR overactivation and inhibition of autophagy in the skeletal muscles of *Mtm1*^–/y^ mice (31, 36). Our study on the *Mtm1*-KI C375S mouse model shows that elevated PI(3)P levels correlate with the overactivation of mTOR in muscle. Specifically, we observed elevated phosphorylation of S6 ribosomal protein and of p70S6K, both downstream targets of mTOR, in the TA muscles of *Mtm1*-KI C375S mice (Figure 7, A and B). Interestingly, no changes were found in the phosphorylation of 4EBP1, another downstream target of mTOR involved indirectly in protein synthesis by inhibition of elF4E protein (Figure 7C). LC3b II protein levels were significantly higher (almost 7-fold increase) in the TA muscle of mutant mice compared with WT animals (Figure 7, D and G). Finally, p62 protein levels, a commonly marker for autophagic flux, were 3-fold higher in the muscles of *Mtm1*-KI C375S mice, while mRNA levels were not affected (Figure 7, E–G). p62 protein is known to accumulate when autophagy is inhibited, leading us to hypothesize that the loss of the MTM1 phosphatase activity results in the decrease of p62 degradation and autophagy. Consistent with this, we also observed increased levels of LC3b II, a marker of autophagosome accumulation, further supporting autophagy disruption. Taken together, these results support that high PI(3)P levels following MTM1 inactivation activate mTOR and inhibit autophagy in muscle.

## Discussion

In this study, we generated and characterized the *Mtm1*-KI C375S mouse, in which the endogenous MTM1 is converted to a phosphatase-dead protein, to distinguish between scaffold and phosphatase-dependent functions and to reveal the role of PI substrate in physiology with a focus on skeletal muscle. We found the MTM1 enzymatic activity and the controlled level of PI(3)P are necessary for survival, motor function, and muscle force, through the regulation of myofiber intracellular organization, organelles positioning, and mTOR activation. While MTM1 is ubiquitously expressed, specific inactivation of its enzymatic activity leads to a progressive myopathy with histological hallmarks similar to XLMTM and to the full MTM1 protein loss in the *Mtm1*^–/y^ mouse, supporting the loss of the phosphatase activity is the main cause of XLMTM.

### MTM1 enzymatic activity and PI(3)P are necessary for myofiber organization and motor functions.

Previous myotubularins mutant mice reported to date were created by approaches removing the whole protein but did not allow to discriminate between the scaffold and phosphatase-dependent functions of these enzymes (30–32, 53, 54). The characterization of the *Mtm1*-KI C375S mouse allowed us, for the first time to our knowledge, to investigate the importance of myotubularins phosphatase activity. Despite its broad tissue expression, MTM1 phosphatase activity and at least its PI(3)P substrate appear mostly essential for skeletal muscle postnatal development and maintenance. In particular, the *Mtm1*-KI C375S mouse displays muscle hypotrophy and muscle weakness, ultimately leading to a shorter survival. Concerning the role of MTM1 on muscle mass, we propose MTM1 modulates muscle mass through the regulation of PI(3)P and the mTOR and autophagy pathways. Our findings are consistent with studies that have demonstrated the correlation of high PI(3)P levels with mTOR overactivation in muscles, particularly in the context of PI3KC2β inactivation, a kinase producing 3-phosphoinositides (44, 45). Specific inactivation of PI3KC2β kinase activity rescued MTM1 loss in mice (45). Along the same lines, Ebner et al. recently proposed that lysosomal PI(3)P and PI(4)P levels regulate mTOR localization and activity (52). In addition, PI(3)P is a main regulator of autophagy via initiation of the phagophore from endoplasmic reticulum and potentially from sorting endosomes, and via the control of autophagosome-lysosome fusion (55, 56). We cannot exclude other minor phosphoinositides regulated by MTM1, as PI(3,5)P_2_ or PI(5)P, might also play a role in addition to PI(3)P. Also, DNM2, which is increased following the inactivation of MTM1 phosphatase activity, was implicated in the maturation and recycling of autolysosomes (57). Overall, the control of mTOR and autophagy through the enzymatic activity of MTM1 is necessary to regulate muscle mass.

Concerning the role of MTM1 on muscle force, which is significantly lower in the *Mtm1*-KI C375S mouse, we hypothesize that PI(3)P regulates myofiber intracellular organization, and especially sarcomere alignment and organelles (mitochondria, triads) position, that are all altered and the main histological defects in the *Mtm1*-KI C375S mouse. Phosphoinositides in general are implicated in membrane trafficking and cytoskeleton organization. DNM2 also binds membranes, actin, and microtubules (58, 59). In addition, BIN1, which is also higher in the *Mtm1*-KI C375S muscles, controls T-tubule formation, binds actin and the microtubules end protein CLIP170, and participates to position the nucleus through binding to the nuclear envelope protein nesprin (60, 61). Defects in these intracellular structures lead to inefficient muscle contraction and to the progressive muscle weakness observed in the *Mtm1*-KI C375S mouse. Overall, the modulation of phosphoinositides by MTM1 enzymatic activity regulates muscle mass and muscle force, while lack of the phosphatase activity triggers muscle hypotrophy and weakness, leading to impaired motor functions and ultimately shorter survival.

### The lack of MTM1 phosphatase activity leads to a moderate XLMTM.

This study alleviates an apparent controversy in the field of myotubular myopathy. Indeed, MTM1 mutations, even most missense, correlated with an absence of the MTM1 protein, making the importance of the enzymatic activity unclear for disease onset and progression. On one hand, the *Mtm1*^–/y^ mouse can be efficiently rescued by decreasing the production of PI(3)P through inactivation of the class II PI 3-kinase PIK3C2B, supporting that the enzymatic activity is key (44, 45, 62). On another hand, the *Mtm1*^–/y^ mouse can be rescued through exogenous expression of phosphatase-dead MTM1 mutants, including C375S mutant, supporting that the scaffolding functions of MTM1 are important (43).

The phenotypes observed in the *Mtm1*-KI C375S mice clearly demonstrate the manifestation and progression of XLMTM, establishing the *Mtm1*-KI C375S model as a faithful model for the disease. All XLMTM hallmarks are present in the model, and the molecular pathways affected in the *Mtm1*^–/y^ were also affected in the *Mtm1*-KI C375S mice (1, 31, 36). Overall, our model confirms that the disease phenotypes result mainly and directly from the loss of enzymatic activity and from phosphoinositides alteration.

Nevertheless, it is important to note that the present data reveal phenotypes in *Mtm1*-KI C375S mice that were less severe compared with those previously observed in the previous well-documented *Mtm1* mouse models lacking MTM1 under various genetic backgrounds and from different teams (30, 33). *Mtm1*-KI C375S mice survived longer, reached higher total body mass, and developed greater muscle force with less profound muscle hypotrophy (with no effect on the mass of gastrocnemius). This supports the idea that the scaffolding functions of MTM1 protein influence the severity and progression of the disease but do not affect the establishment of the phenotype. The fact that exogenous expression of MTM1 phosphatase-dead mutants rescued most, albeit not all, XLMTM-like phenotypes in the *Mtm1*^–/y^ mouse may be due to the effect of scaffolding functions combined to a dominant negative effect on PI(3)P functions (43). Indeed, the exogenous phosphatase-dead MTM1 may still bind its PI(3)P substrate, preventing the deleterious effect of PI(3)P increase in the disease and mimicking the effect of PI(3)P downregulation obtained by PI 3-kinase inhibition. The reported rescue of mitochondrial defects by exogenous expression of MTM1 phosphatase-dead mutants in *Mtm1*^–/y^ mice in contrast to the presence of these defects in *Mtm1*-KI C375S mice could be explained by the later timing of exogenous mutant expression (43). These findings indicate that reducing symptom severity in patients with XLMTM could be achieved by mimicking the protein interactions of MTM1, highlighting this approach as a potential direction for future therapy discovery.

## Methods

### Sex as a biological variable.

Our study exclusively examined male mice as the *Mtm1* gene is on the X chromosome.

### Animals.

The *Mtm1-*KI C375S mouse line was established in the Biomedical Sciences Research Centre “Alexander Fleming” in Greece. CRISPR-Cas9 technology was used to insert 3-point mutations in the *Mtm1* gene, including codon TGC corresponding to Cys375 to TCG, with the desired mutation resulting in replacing the catalytic cysteine with a serine, and the silent mutation creating an XhoI restriction site for genotyping purposes. Additionally, a silent mutation (T>C) was introduced upstream to the PAM sequence. The crRNA with the sequence GTACTTGTGCACTGCAGTGACGG and the single-stranded oligodeoxynucleotides (ssODN) with sequence 5′-ACCGGTGCCATTCAAGTGGCAGACCAAGTGTCTTCAGGAAAGAGCTCGGTACTTGTGCACTCGAGCGACGGATGGGACAGGACCGCTCAGCTGACATCCTTGGCCATGCTGATGTTGGAC-3′ were used. The line was on a mixed background, 50% C57BL/6J 50% CBA. Mice were housed in the Institut Clinique de la souris animal facility in ventilated cages under controlled conditions of temperature and humidity with 12-hour light/dark cycles, where they had access to food and water ad libitum. Only male littermates were analyzed in this study as the Mtm1 gene is on the X chromosome and as females did not show an obvious phenotype. All experiments were performed on 8-week-old mice, unless otherwise specified.

### Genotyping.

For genotyping purposes, finger biopsies were used. Genotyping primers were 5′-AGACGGAATGGGAGGTGGT-3′ (forward) and 5′-GGCTTCATTACACTGCTCTTGA-3′ (reverse). The PCR products were digested with the XhoI enzyme for 30 minutes at 37°C. Digestion resulted in a single band with 631 bp size for the WTs and extra bands at 461 and 170 bp for the mutant mice, after electrophoresis of the PCR products. Additionally, the PCR product from one WT and one mutant mouse was subjected to Sanger sequencing for confirmation.

### RNA extraction and quantitative PCR.

TA and GA muscles were snap frozen in liquid nitrogen and stored at –80°C. Fragments of the muscle samples were used for RNA extraction by lysis in TRl Reagent (Molecular Research Center, #TR 118) and homogenization with a Precellys Evolution Touch homogenizer (Bertin Technologies). For reverse transcription, SuperScript IV Reverse Transcriptase (Thermo Fisher Scientific, #18090050) and 1 μg of RNA from the samples were used. For the quantification of mitochondrial to nuclear DNA ratio in TA muscles, DNA was isolated from pieces of the TA muscles using lysis buffer containing 100 mM Tris-HCl, 20 mM NaCl, 0.2% SDS, 5mN EDTA and Proteinase K 100 mg/mL. Samples were incubated at 60°C for 1 hour and final pellets were diluted in TE buffer containing 10 mM Tris-HCl and 1mM EDTA. cDNAs and DNAs were amplified with SYBR Green I Master (Roche, #04887352001) in a LightCycler 480 (Roche). Three technical replicates were utilized for the qPCR. The following primers were used: *Mtm1* forward: 5′-TGAGTGAGACTGTCCCTCGG-3′, *Mtm1* reverse: 5′-TGGGGCCATTGAAAGGACAT-3′, *Nd1* forward: 5′-AAGTTGATCGTAACGGAAGC-3′, *Nd1* reverse: 5′-CCCATTCGCGTTATTCTT-3′, *Bin1* forward: 5′-CCTGTCTCGCTGCTTGAGAAA-3′, *Bin1* reverse: 5′-CTCGAACACCTTCTGGGCTT-3′, *Dnm2* forward: 5′-ACCCCACACTTGCAGAAAAC-3′, *Dnm2* reverse: 5′-CGCTTCTCAAAGTCCACTCC-3′, *p62* forward: 5′-TGTGGAACATGGAGGGAAGA-3′, *p62* reverse: 5′-TGTGCCTGTGCTGGAACTT-3′, *Mtmr1* forward: 5′-CCTCAACAAGCATGCTTTTCCTCT-3′, *Mtmr1* reverse: 5′-CTAGTTGGCACAACAATGATGGCA-3′, *Mtmr2* forward: 5′-GACTCACTGTCCAGTGCTTC-3′, *Mtmr2* reverse: 5′-TTCTGCTAACTTGTTTGCCTCCC-3′, *Mtmr13* forward: 5′-TCTGAAGTACAGTTACCCGTATATC-3′, *Mtmr13* reverse: 5′-GATTACATCTAAAAGCTCATGGACATC-3′, *Mtmr5* forward: 5′-GAAGTGTTCAGGAATAGCCTTGG-3′, *Mtmr5* reverse: 5′-AGCCAATGGGACGGTACATGT-3′, *PI3KC2b* forward: 5′-CCGACTGGCTACAAAAACACAAC-3′, *PI3KC2b* reverse: 5′-GCCAGCACAAGAGTAGATGAAGTT-3′, *Myh2* forward: 5′-ATCCAAGTTCCGCAAGATCC-3′, *Myh2* reverse: 5′-TTCGGTCATTCCACAGCATC-3′, *Myh4* forward: 5′-AGACAGAGAGGAGCAGGAGAGTG-3′, *Myh4* reverse: 5′-CTGGTGTTCTGGGTGTGGAG-3′, *Myh1* forward: 5′-ATGAACAGAAGCGCAACGTG-3′, *Myh1* reverse: 5′-AGGCCTTGACCTTTGATTGC-3′, *Myh7* forward: 5′-CTACAGGCCTGGGCTTACCT-3′, *Myh7* reverse: 5′-TCTCCTTCTCAGACTTCCGC-3′, *Rpl27* forward: 5′-AAGCCGTCATCGTGAAGAACA-3′, *Rpl27* reverse: 5′ -CTTGATCTTGGATCGCTTGGC-3′, *Stau1* forward: 5′-AAGAAGGTGGCCAAGCGTAA-3′, *Stau1* reverse: 5′-ATTTGAGTGCTGGCTTGGCA-3′. *Rpl27* or *Stau1* were used as housekeeping genes for standardization.

### Protein extraction and Western blotting.

TA and GA muscles were snap frozen in liquid nitrogen and stored at –80°C. Fragments of the muscle samples were lysed in RIPA buffer supplemented with 1 mM PMSF, 1 mM DTT, 5 mM sodium fluoride, 1mM sodium orthovanadate, and complete mini EDTA-free protease inhibitor cocktail (Roche Diagnostics, 11 836 170 001) by incubation for 15 minutes on ice. The tissues were then homogenized using a Precellys Evolution Touch homogenizer (Bertin Technologies) to ensure thorough cell lysis and protein extraction. For some of the experiments, subcellular fractionation of the muscle samples into nuclear, cytosolic, and mitochondrial fractions was performed according to the protocol previously described by Dias et al. (63). Protein concentration in the samples was determined using the DC protein Assay kit (Bio-Rad, 5000113, 5000114, 5000115) according to manufacturer’s instructions. 15 μg of protein were denaturated for 5 minutes at 95°C in 5x Lane Marker Reducing Buffer (Thermo Fisher Scientific) and separated in 10% SDS-PAGE homemade gel or in 4–15% mini-PROTEAN TGX precast protein gel (Bio-Rad, #4561086). PageRuler Plus Prestained Protein Ladder (Thermo Fisher Scientific, #26619) was also loaded on each gel for reference. Proteins were then transferred to nitrocellulose membranes using a Trans-Blot Turbo Transfer System (Bio-Rad, #1704150). Membranes were stained with Ponceau S to verify protein transfer and to normalize protein loading across samples. Membranes were then blocked in Tris-Buffered Saline with 0.1% Tween-20 (TBS-T) and 5% nonfat dry milk for 1 hour at room temperature and subsequently incubated with primary antibodies overnight at 4°C. The next day the membranes were washed in TBS-T and incubated for 1 hour at room temperature with secondary antibodies. Following the final washes in TBS-T, membranes were visualized with an Ammersham Imager 600 (GE Healthcare Life Sciences) with prior addition of SuperSignal West Pico PLUS Chemiluminescent Substrate (Thermo Fisher Scientific, #34577). For the quantification of the band intensity the Fiji software was used. The uncropped pictures of the blots are shown in Supplemental Figure 3. The primary and secondary antibodies used were: anti-MTM1 (rabbit, 1:700, homemade targeting the C-terminal end, #2827) (38), anti-BIN1 (rabbit, 1:1000, R3623; homemade targeting the SH3 domain, #3623), anti-DNM2 (rabbit 1:1000, homemade targeting the proline-rich domain, 2865), anti-prohibitin (rabbit 1:1000, abcam, #ab28172), anti-OXPHOS (mouse 1:1000, Thermo Fisher Scientific, #45-8099), anti-p62 (mouse, 1:1000, Novus Biologicals, #H00008878-M01), anti-phospho-S6 Ribosomal Protein (Ser235/236) (rabbit, 1:1000, Cell Signaling Technology, #2211), anti-S6 Ribosomal Protein (rabbit, 1:1000, Cell Signaling Technology, #2217), anti-phospho-p70S6 (mouse, 1:1000, Cell Signaling Technology, #9206), anti- p70S6 (rabbit, 1:1000, Cell Signaling Technology, #2708), anti-phospho-4EBP1 (rabbit, 1:1000, Cell Signaling Technology, #9459), anti-4EBP1 (rabbit, 1:1000, Cell Signaling Technology, #9644), anti-LC3 (rabbit, 1:1000, Cell Signaling Technology, #9206), anti-GAPDH (mouse, 1:1000, MERCK, #MAB374), anti-Lamin A (rabbit, 1:800, abcam, ab26300), anti-TOMM20 (rabbit, 1:1000, abcam, #ab78547), horseradish peroxidase-coupled goat anti-rabbit (goat, 1:10000, #112-036-04), and horseradish peroxidase-coupled goat anti-mouse (goat, 1:10000, #115-036-068).

### PI(3)P mass ELISA.

Gastrocnemius muscles were snap frozen in liquid nitrogen and stored at –80°C. To extract the lipids, muscle pieces were homogenized using a Precellys Evolution Touch homogenizer (Bertin Technologies) in 1 mL ice-cold 5% trichloroacetic acid with 1 mM EDTA. The homogenized sample was further diluted in 2 more mL of the same buffer, agitated for 30 seconds and centrifuged for 5 minutes at 900*g*. The supernatant was discarded and the washing step was repeated another time. The pellet was then resuspended in 3 mL MeOH:CHCl_3_ and agitated for 10 minutes for the extraction of neutral lipids. After centrifugation for 5 minutes at 900*g* and a repetition of the neutral lipid extraction step, acidic lipids were extracted by resuspension of the pellet in MeOH: CHCl_3_: 12 N HCl (80:40:1) and an agitation step of 25 minutes. The samples were then centrifuged and the supernatant was mixed with 0.75 mL CHCl_3_ and 1.35 mL 1N HCl. A 30-second agitation followed by centrifugation allowed the collection of the lower organic phase from the sample. The organic phase containing the lipids was then dried in a vacuum drier for 1 hour at room temperature. Subsequently, PI(3)P levels were determined in the samples using the PI(3)P mass ELISA kit (Echelon Bioscience Inc, #K-3300) according to manufacturer’s instructions.

### Quantification of PI(3)P by mass spectrometry.

Phosphoinositides were extracted as described in study by Clark et al. (64) then analyzed as described in study by Li et al. (65). Briefly, 2mg of TA muscle was lysed in lysing matrix S tubes (MP Biomedical) containing 750 μL CHCl_3_/MeOH/1 M HCl (v/v/v: 10/20/1). Phosphoinositides were extracted with a 2-phase system in which the lower organic phase contained lipids, methylated by (Trimethylsilyl)diazomethane, and separated on Daicel Chiralpak IC-U column for analysis on the LC-QQQ triple quadrupole mass spectrometer (LCMS-8060 Shimadzu) using LabSolutions LCMS software. Offline analysis was performed using LabSolutions Insight LCMS software. Data were quantified as ratio area sample/area internal standard.

### Mouse in vivo phenotyping.

Starting at 4 weeks of age, mice were monitored daily for severe clinical signs and their body mass was measured. Once a week, the hanging test was performed and the Disease Severity Score (DSS) was assessed. For the hanging test, the mice were suspended upside down on a cage grid for a maximum of 60 seconds. The time of fall was recorder. The test was repeated 3 times per mouse with a 5-minute recovery interval between each trial. The DSS was calculated using a scoring system that evaluated their body mass, their performance on the hanging test, the presence of kyphosis and walking difficulties. Details on the scoring system can been found on Supplemental Table 1. At 8 weeks of age, body composition analysis was performed to determine fat content, lean tissues and free body fluid content. This was achieved using Nuclear Magnetic Resonance (NMR) with the Minispec+ analyzer (LF110-Bruker). Measurements were conducted during light period on conscious, fed mice.

### In situ muscle force measurements.

Muscle contractile properties were assessed by measuring the contraction of the TA muscle following stimulation of the sciatic nerve. The Complete 1300A mouse Test System (Aurora Scientific) was utilized. Eight-week-old mice were anesthetized by administering of Domitor (2 mg/kg)/Fentanyl (0.28 mg/kg), Diazepam (8 mg/kg), and finally Fentanyl alone (0.28 mg/kg) via i.p. injections. A small incision was made at the lateral side of the leg to expose the sciatic nerve. The distal tendon of the TA muscle was carefully excised and securely tied to the isometric transducer using a nonelastic suture, while the knee and foot were fixed to prevent movement. The sciatic nerve was then stimulated at frequencies ranging from 2 to 150 Hz. Specific force values were calculated by dividing the absolute force by the mass of the TA muscle.

### Histology.

TA and GA muscles were frozen in liquid nitrogen-cooled isopentane and stored at –80°C. The samples were cut at 8 μm transversal sections and stained with hematoxylin and eosin (H&E) or with succinate dehydrogenase (SDH). The stained samples were then imaged with a Nanozoomer 2HT slide scanner (Hamamatsu) and each myofiber was individually segmented using the Celllpose algorithm (66). To calculate the MinFeret diameter and to manually assess the number of fibers with mispositioned nuclei, Fiji was used. Classification of myofibers regarding SDH staining was done manually with Fiji (67).

### Immunofluorescence staining.

TA muscles were frozen in liquid nitrogen-cooled isopentane and stored at –80°C. The samples were cut at 8 μm transversal sections. The fiber type immunofluorescence was performed in nonfixed and nonpermeabilized transversal sections. Slides were blocked for 1 hour with 3% BSA in PBS. Antibodies were diluted in 3% BSA in PBS and incubation was performed at 4°C overnight for the primary and at room temperature for 1 hour for the secondary. In the buffer including the secondary antibodies, Wheat Germ Aglutinin Alexa 647 (Invitrogen, # W32466) was also included in a 1:200 dilution. Primary antibodies were anti-myosin type I, BA-D5 (DSHB, AB_2235587), anti-myosin type IIa, sc-71 (DSHB, AB_2147165) and anti-myosin type IIb, BF-F3 (DSHB, AB_2266724), all diluted 1:50. Secondary antibodies were goat anti-mouse IgG2b Cy3 (Jackson ImmunoResearch, #115-165-207), goat anti-mouse IgG1 Alexa 488 (Jackson ImmunoResearch, #115-545-205) and goat anti-mouse IgMμ DyLight 405 (Jackson ImmunoResearch, #115-475-075), all diluted 1:100. Images were acquired using the Axioscan 7 digital slide scanner (ZEISS).

### Electron microscopy.

Pieces from the TA muscle were fixed in 2.5% paraformaldehyde (PFA, Electron Microscopy Sciences), 2.5% glutaraldehyde (Electron Microscopy Sciences) and 50 mM CaCl2 (Sigma-Aldrich) in cacodylate buffer (0.1M, pH 7.4, Sigma-Aldrich). The samples were then further fixed using 1% osmium tetroxide in cacodylate buffer (0.1M) for 1 hour at 4°C. For dehydration, graded alcohol and propylene oxide were used for 30 minutes each. The samples were then embedded in Epon 812, cut at ultrathin sections (70nm) and contrasted with uranyl acetate and lead citrate. Observation was done under a Philips CM12 electron microscope equipped with a Gatan OneView Camera (Gatan).

### ATP production and citrate synthase (CS) activity.

ATP levels in TA muscle samples were measured using the ATP Assay Kit (Colorimetric/Fluorometric) (abcam, #ab83355) following the manufacturer’s instructions. Briefly, 10mg of TA muscle were washed in cold PBS and homogenized in 100 μL of the assay buffer provided by the kit, using Dounce homogenizer on ice for 10 passes. The homogenates were then deproteinized using the TCA deproteinization sample preparation kit (abcam, #ab204708) according to manufacturer’s protocol. ATP content was subsequently quantified using the colorimetric assay procedure provided in the kit.

Citrate synthase activity in TA muscles was measured using the Citrate Synthase Assay Kit (abcam, #ab239712) according to manufacturer’s instructions. In short, 10mg of TA muscle were washed in cold PBS and homogenized in 100 μL of the assay buffer provided by the kit using a Precellys Evolution Touch homogenizer (Bertin Technologies). Protein concentration in the samples was determined using the DC protein Assay kit (Bio-Rad, #5000113, 5000114, 5000115) according to manufacturer’s instructions. Samples were transferred in a 96-well plate (dilution factor 10) and reaction mix and background control were added to respective wells. CS activity was measured on a microplate reader in kinetic mode at 412 nm, by following the initial reaction rate of GSH and subsequently CS and CoA levels. Two time points were selected and CS activity was calculated according to the provided formula and normalized to protein levels.

### Blood collection and analysis.

For the collection of blood and plasma, EDTA-coated Microvette K3E tubes (Sarstedt, #20.1341.100) were used. Samples were kept on ice until analysis with the Element HT5 Hematology Analyzer (SCIL) to determine blood cell counts. For the plasma isolation, samples were centrifuged at 4°C for 15 minutes at 2000*g*. Plasma was transferred to new tubes and stored at –80°C. Analysis was performed using an AU480 Chemistry Analyzer (Beckman Coulter).

### Statistics.

All data were verified for normal distribution using the Shapiro-Wilk test. When normality was confirmed, unpaired parametric 2-tailed *t* test was performed. Welch’s corrections were used when the variances were found unequal. For data not fitting the normal distribution, the Mann-Whitney *U* test was used. Two-way ANOVA was performed on stimulation frequency to analyze force values during the force frequency protocol, on fiber size to compare percentage of fibers for the H&E staining analysis, on the DSS scores and on the hanging test results, followed by Šídák multiple-comparison test. Statistical significance was considered for *P* < 0.05. Graphs demonstrate individual points, with additional lines indicating the mean value ± SEM. Statistical analyses were performed in GraphPad Prism 10 software.

### Study approval.

All animal experiments were performed in accordance with French and European legislations and approved by the institutional ethics committee (project no. APAFIS#35021-2022012717476388).

### Data availability.

The authors declare that all data supporting the findings of this study are available in the article or its supplemental material. Values for all data points in graphs are reported in the Supporting Data Values file.

## Author contributions

JL conceived the project. FMF and GC performed molecular experiments. FMF, DR, and CK performed in vivo experiments. JL and BP provided funding and supervised the work. FMF and JL wrote the manuscript.

## Funding support

Interdisciplinary Thematic Institute IMCBio+, as part of the ITI 2021-2028 program of the University of Strasbourg, CNRS and Inserm.

- IdEx Unistra (ANR-10-IDEX-0002) under the framework of the France 2030 Program.
- SFRI-STRAT’US project (ANR-20-SFRI-0012) under the framework of the France 2030 Program.
- EUR IMCBio (ANR-17-EURE-0023) under the framework of the France 2030 Program.

## Supplementary Material

Supplemental data

Unedited blot and gel images

Supplemental video 1

Supporting data values

## Version 1 10/14/2025

In-Press Preview

## Version 2 11/24/2025

Electronic publication
