# Supplementary material for: Lack of myotubularin phosphatase activity is the main cause of X-linked myotubular myopathy
Source: JCI Insight. 2025 Oct 14;10(22):e189286. doi: 10.1172/jci.insight.189286 (PMC12643485; doi:10.1172/jci.insight.189286)
Supplement: Supplemental data [file jciinsight-10-189286-s147.pdf]

Supplemental Figure 1

A

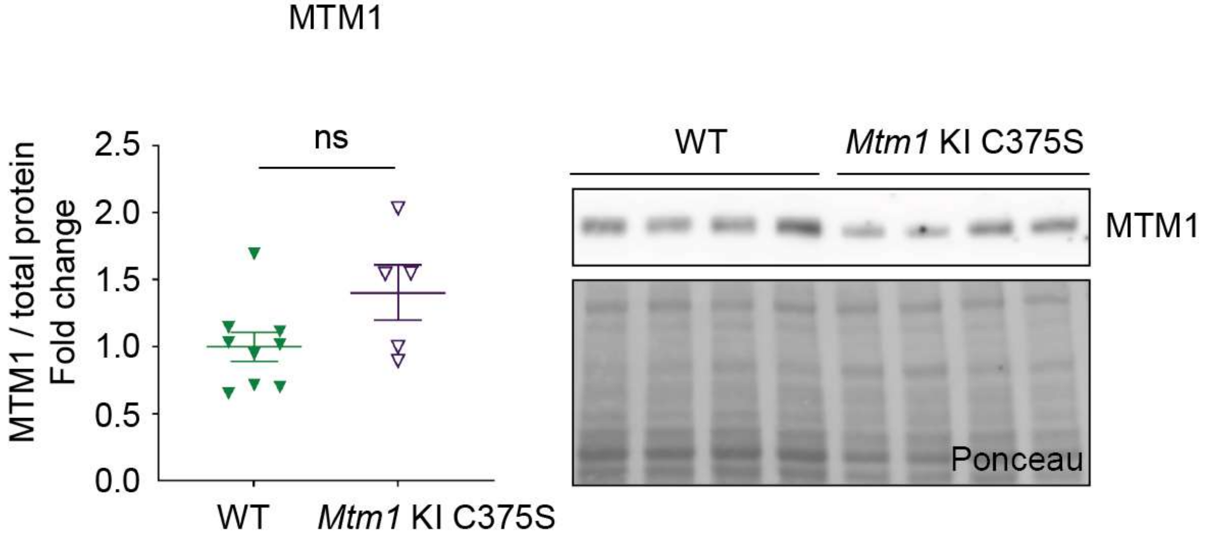

B

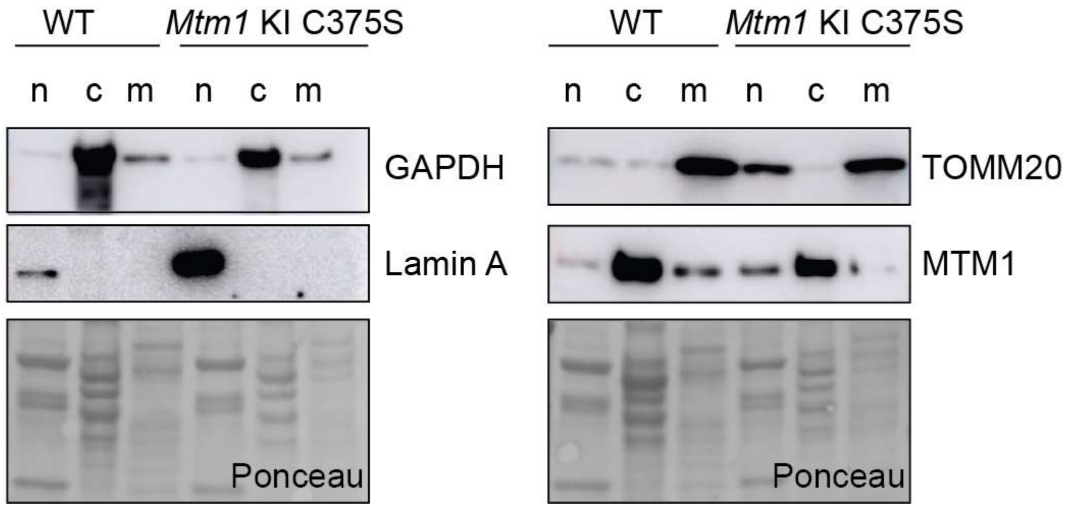

Supplemental Figure 2

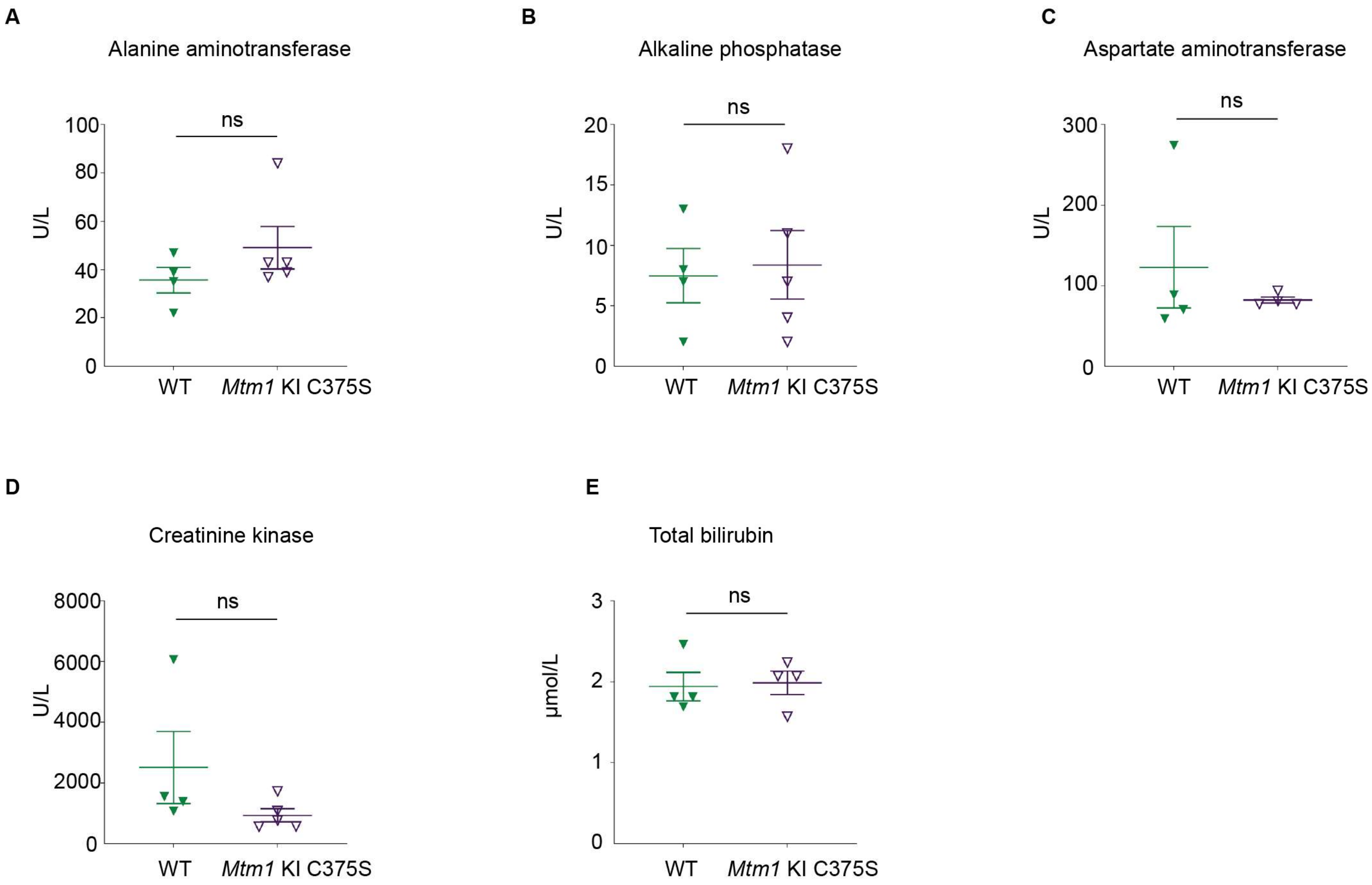

Supplemental Figure 3

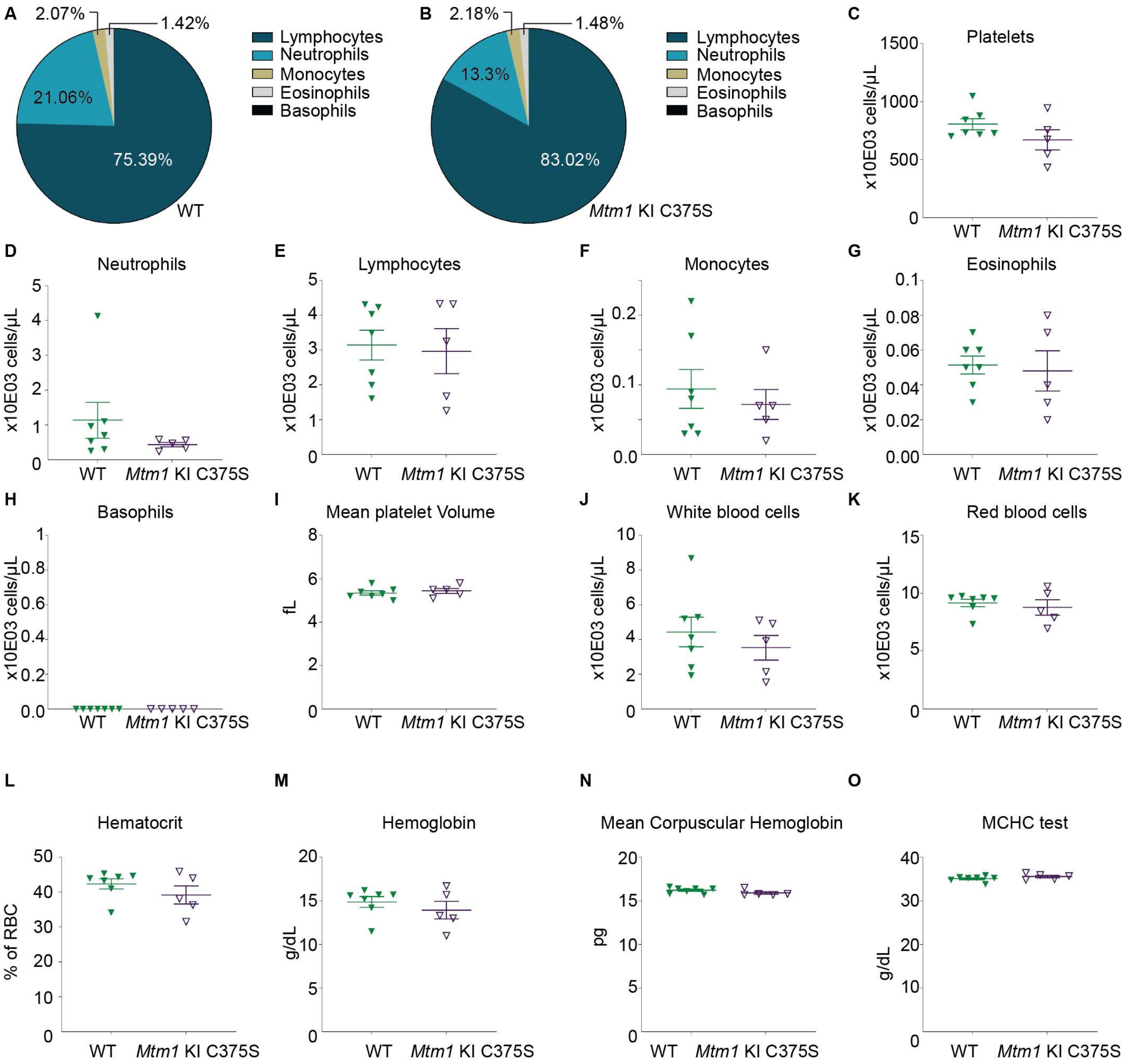

Supplemental Figure 4

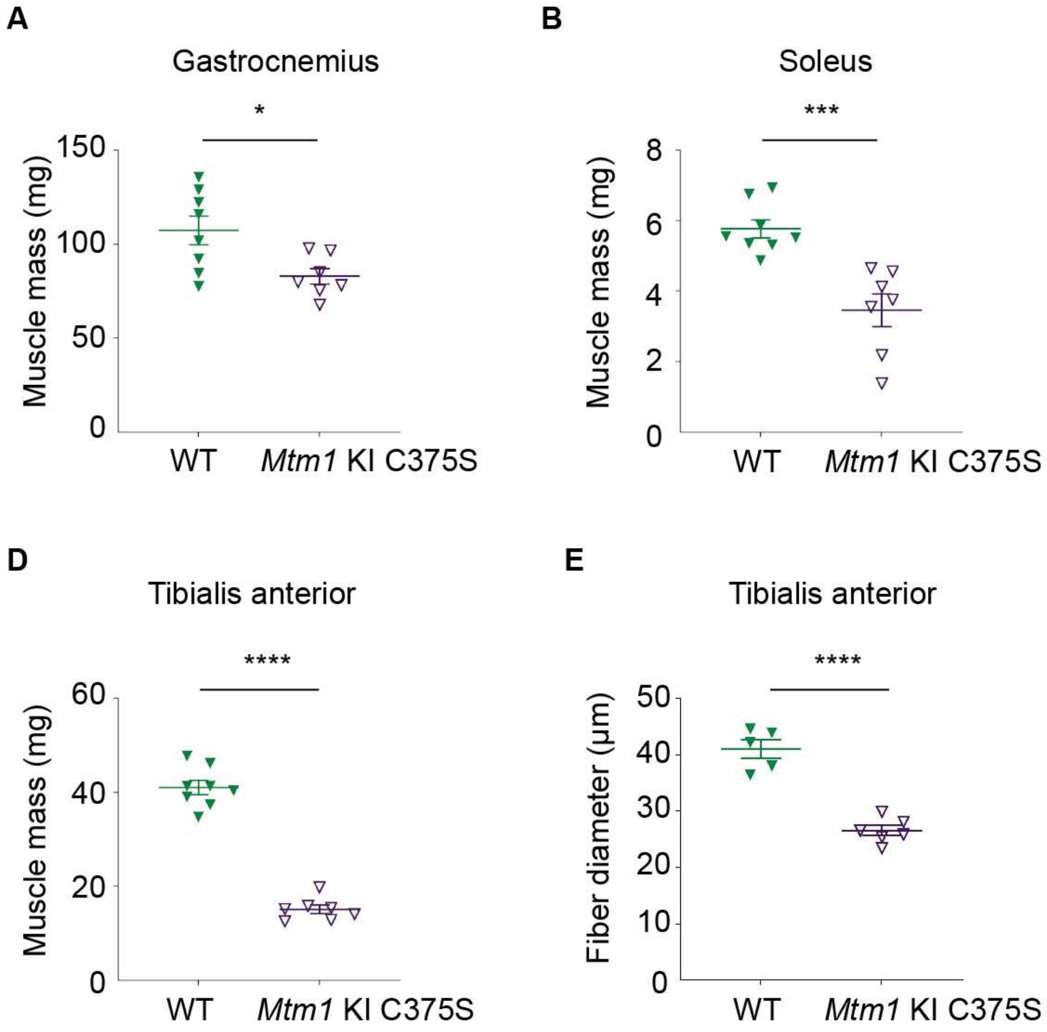

Supplemental Figure 5

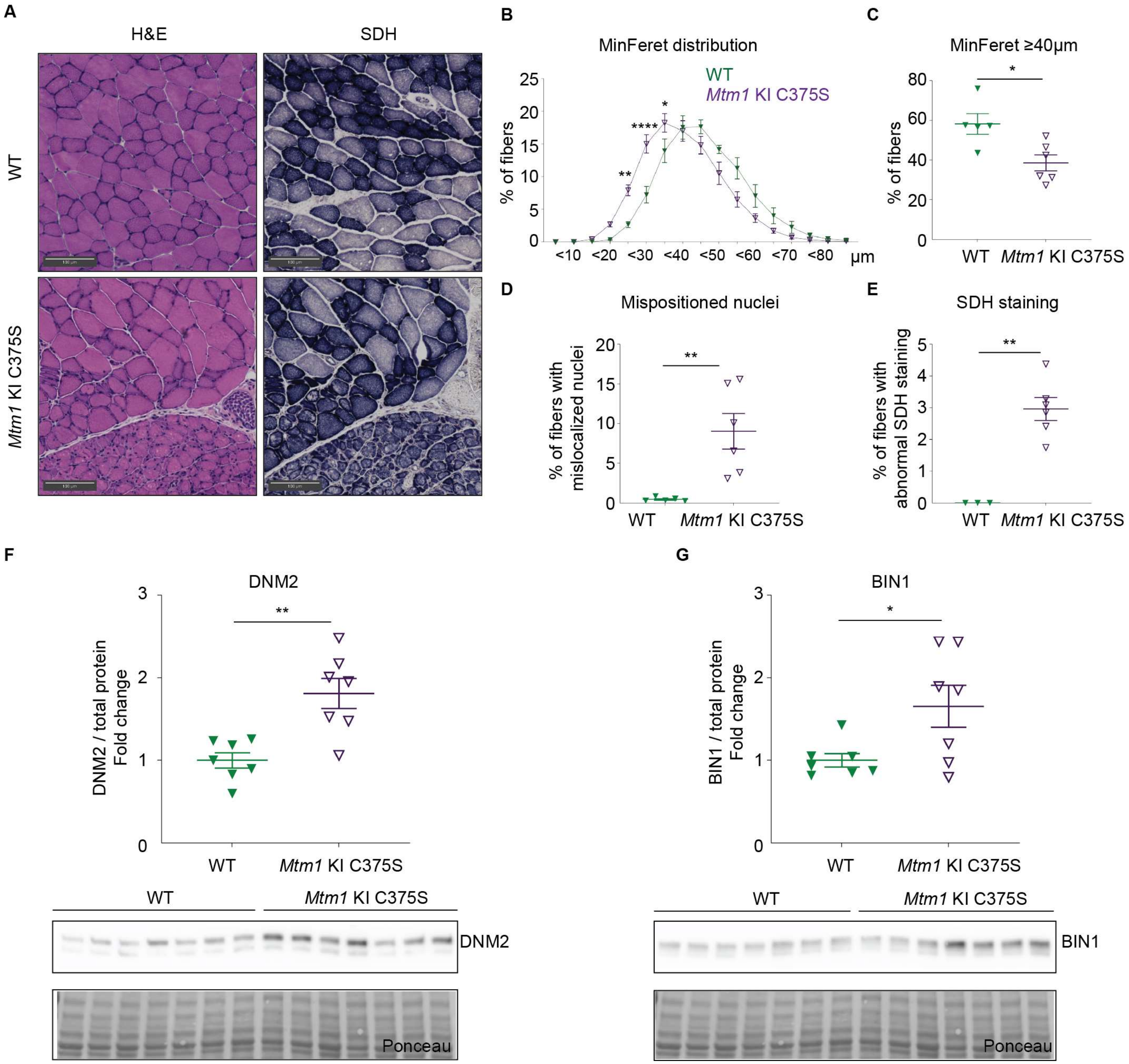

Supplemental Figure 6

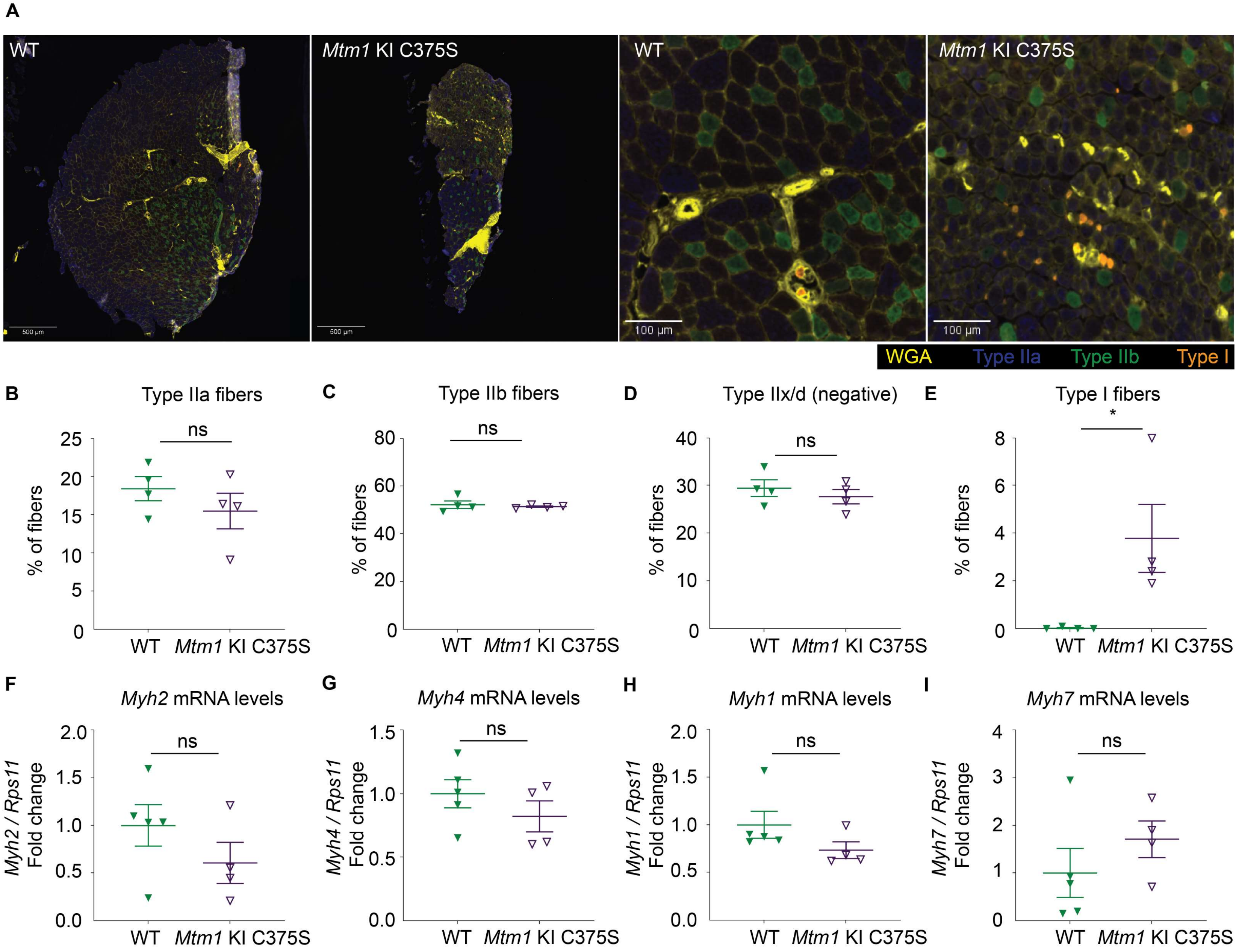

Supplemental Figure 7

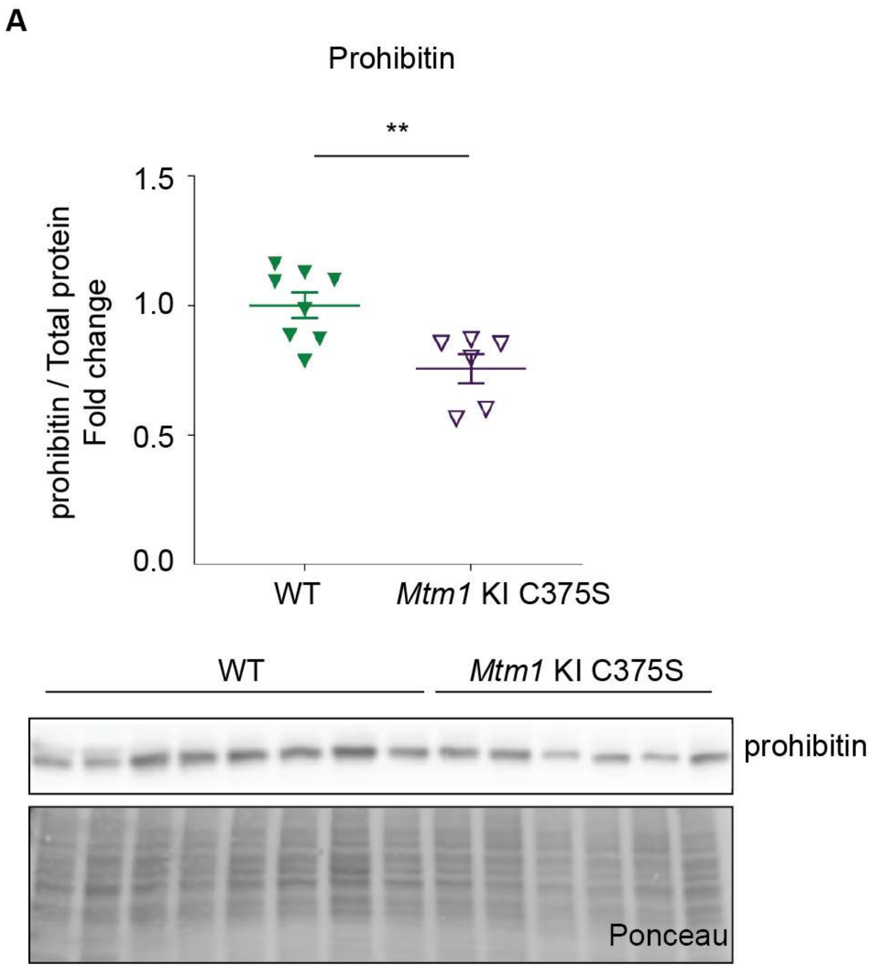

Supplemental Figure 8

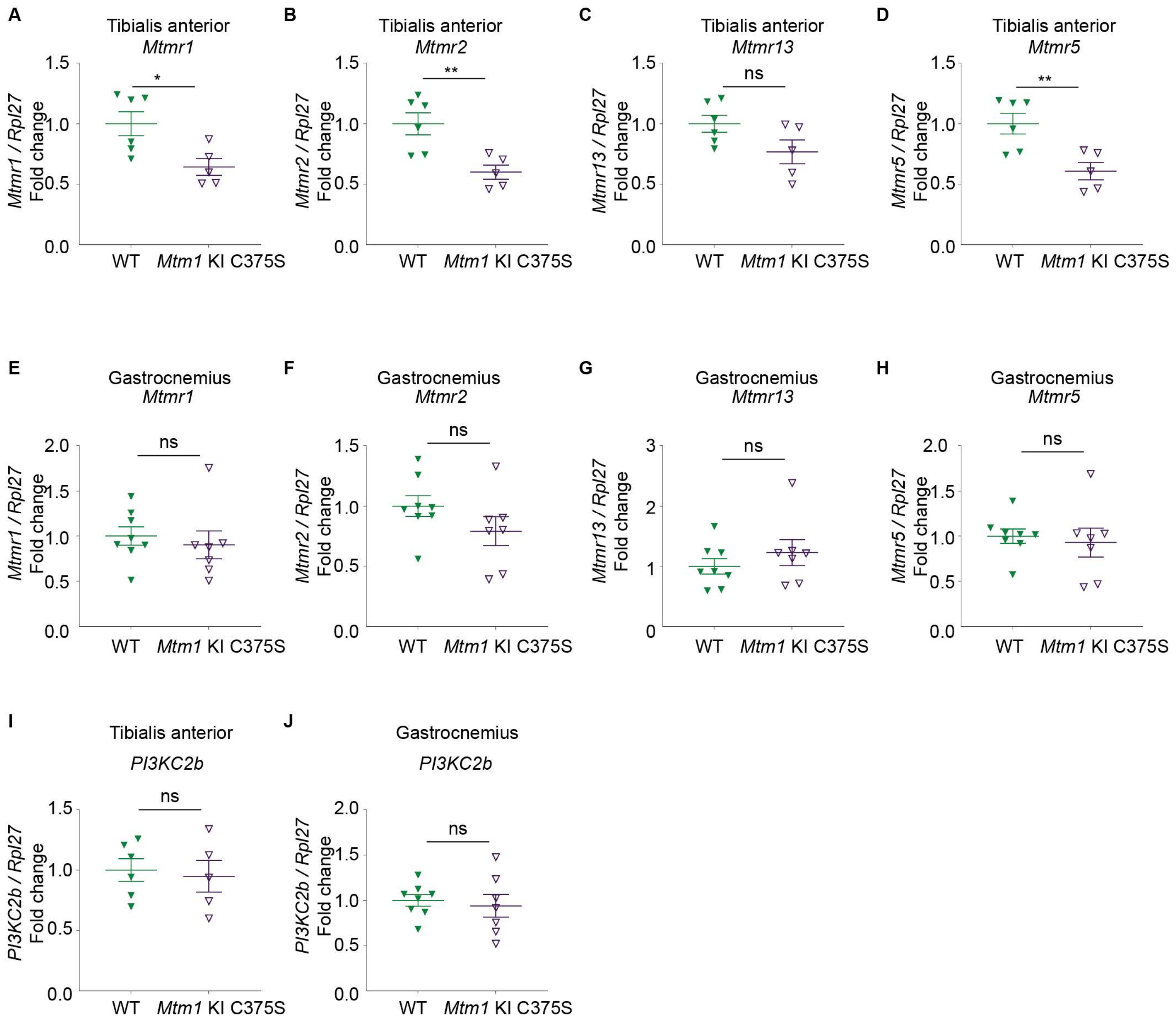

Supplemental Table 1

Disease scoring factors	Description	Score
Body mass	**Score 0-1**<br>Difference in body mass from average of wildtype littermates	**0** for 0-1g<br>**0.5** for >1-2g<br>**1** for >2g
Hanging test ability	**Score 0-1**<br>Representing 0 to 60 seconds hanging time	**0** for 60s<br>**0.5** for 5-59s<br>**1** for <5s
Kyphosis	**Score 0-1**<br>Curvature of the spine	**0** for no curvature<br>**0.5** for mild curvature<br>**1** for severe curvature
Walking difficulties	**Score 0-1**<br>Ability to use hind limbs	**0** for normal use<br>**0.5** for splayed use<br>**1** for loss of use
Maximum score	**4**	
