# Supplementary material for: Lack of myotubularin phosphatase activity is the main cause of X-linked myotubular myopathy
Source: JCI Insight. 2025 Oct 14;10(22):e189286. doi: 10.1172/jci.insight.189286 (PMC12643485; doi:10.1172/jci.insight.189286)

Full unedited gel for figure 1C

Genotyping primers were 5'-AGACGGAATGGGAGGTGGT-3' (forward) and 5'-GGCTTCATTACACTGCTCTTGA-3' (reverse)

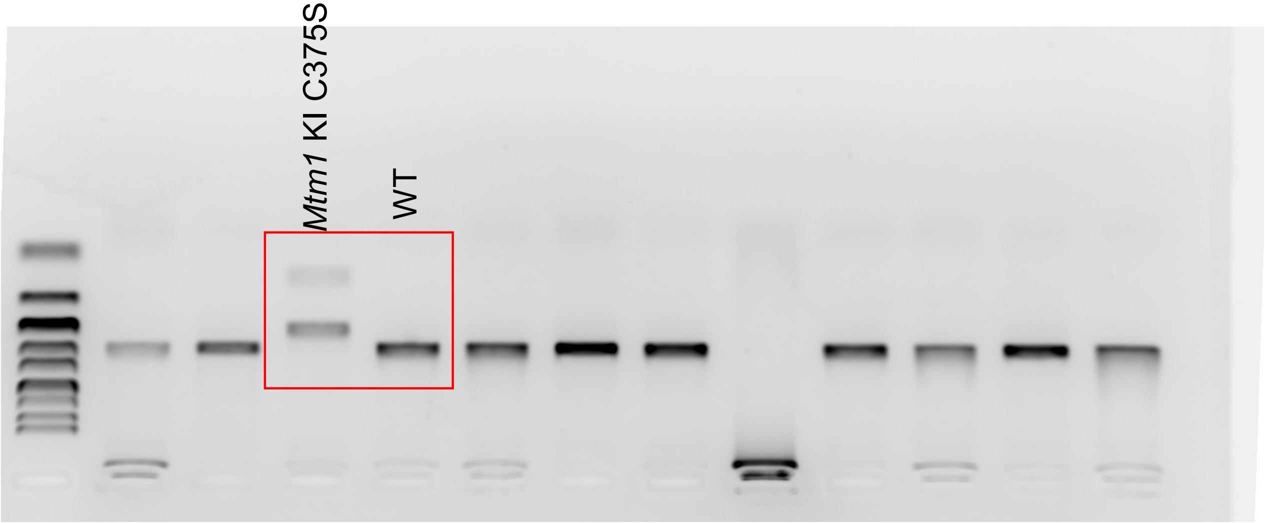

Full unedited blot for figure 1E

Primary antibody: anti-MTM1 (rabbit, 1:700, homemade targeting the C-terminal end, #2827)

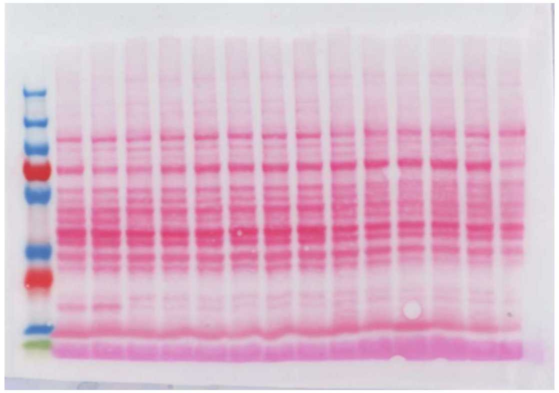

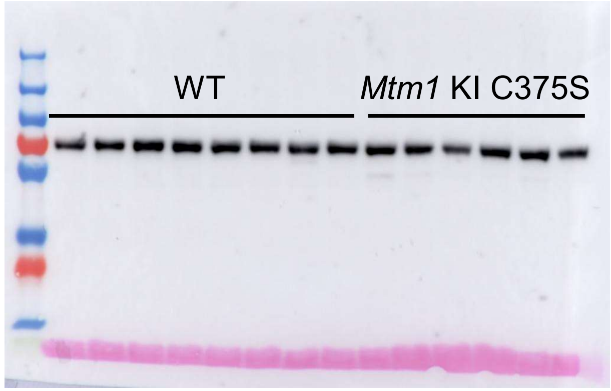

Full unedited blot for figure 5D

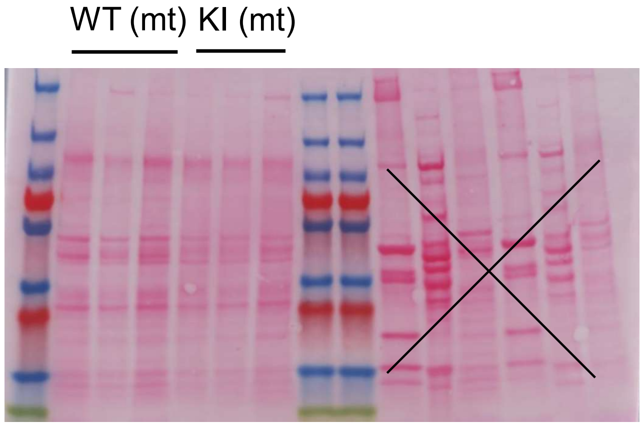

<u>Primary antibody:</u> anti-OXPHOS (mouse 1:1000, Thermofisher Scientific, #45-8099)

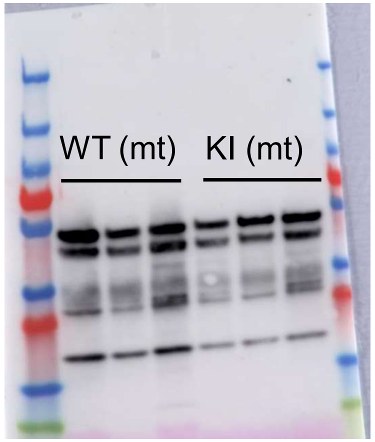

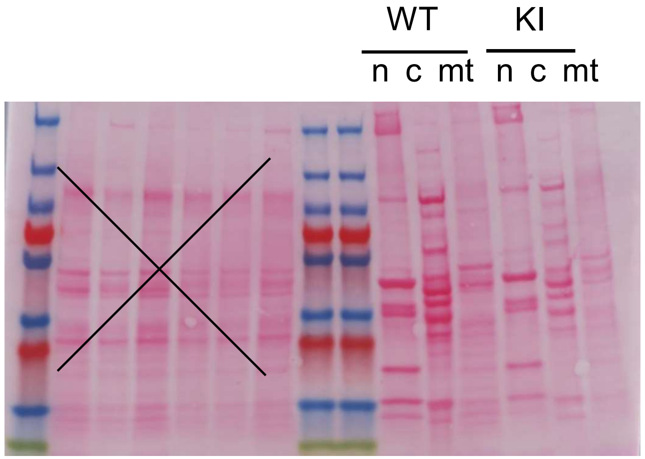

<u>Primary antibody:</u> anti-GAPDH (mouse, 1:1000, MERCK, #MAB374)

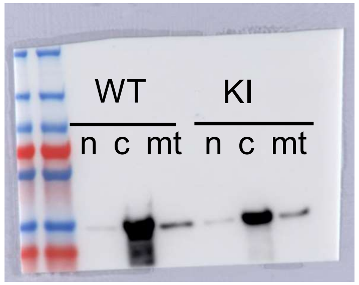

<u>Primary antibody:</u> anti-Lamin A (rabbit, 1:800, abcam, ab26300)

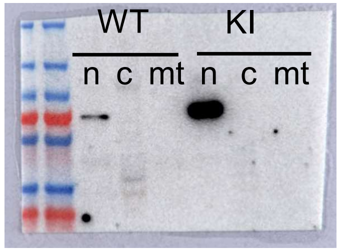

Full unedited blot for figure 6A

<u>Primary antibody:</u> anti-DNM2 (rabbit 1:1000, homemade targeting the proline-rich domain, 2865)

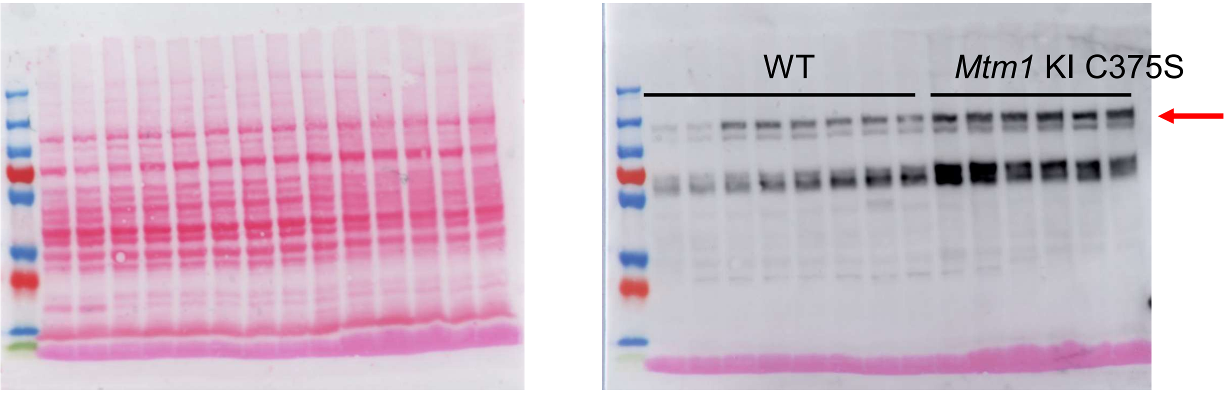

Full unedited blot for figure 6C

<u>Primary antibody:</u> anti-BIN1 (rabbit, 1:1000, R3623; homemade targeting the SH3 domain, #3623)

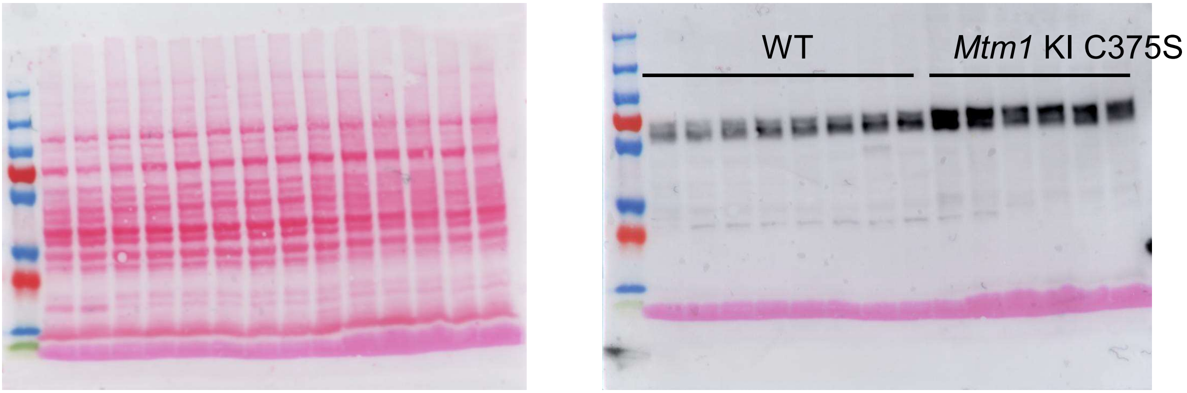

Full unedited blot for figure 7A

Primary antibody: anti-phospho-S6 Ribosomal Protein (Ser235/236) (rabbit, 1:1000, Cell Signaling Technology, #2211)

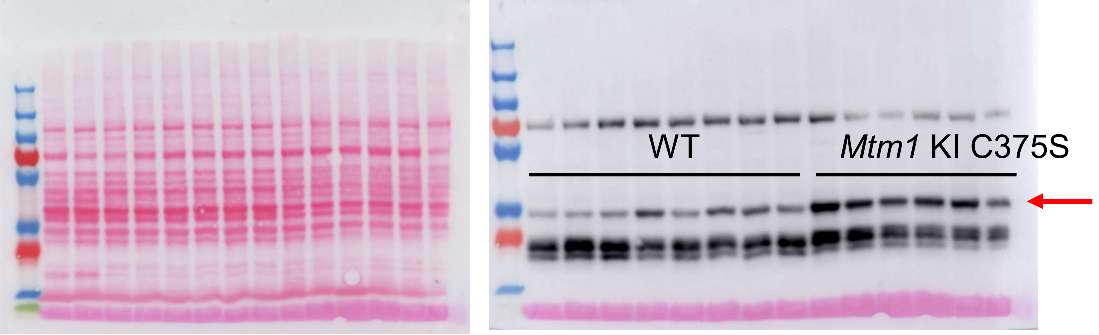

Primary antibody: anti-S6 Ribosomal Protein (rabbit, 1:1000, Cell Signaling Technology, #2217)

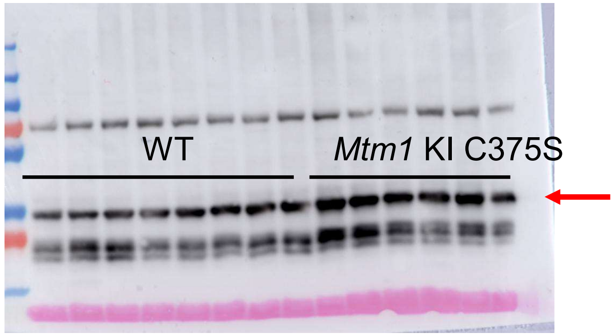

Full unedited blot for figure 7B

Primary antibody: anti-phospho-p70S6 (mouse, 1:1000, Cell Signaling Technology, #9206)

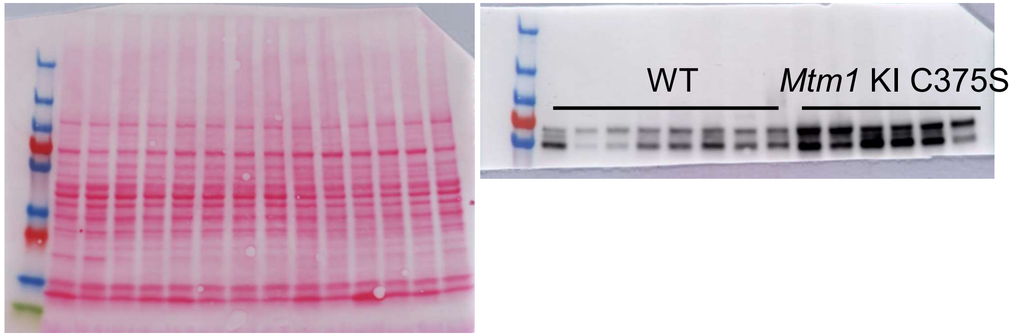

Primary antibody: anti- p70S6 (rabbit, 1:1000, Cell Signaling Technology, #2708)

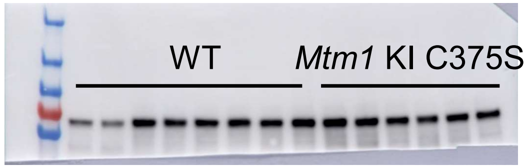

Full unedited blot for figure 7C

Primary antibody: anti-phospho-4EBP1 (rabbit, 1:1000, Cell Signaling Technology, #9459)

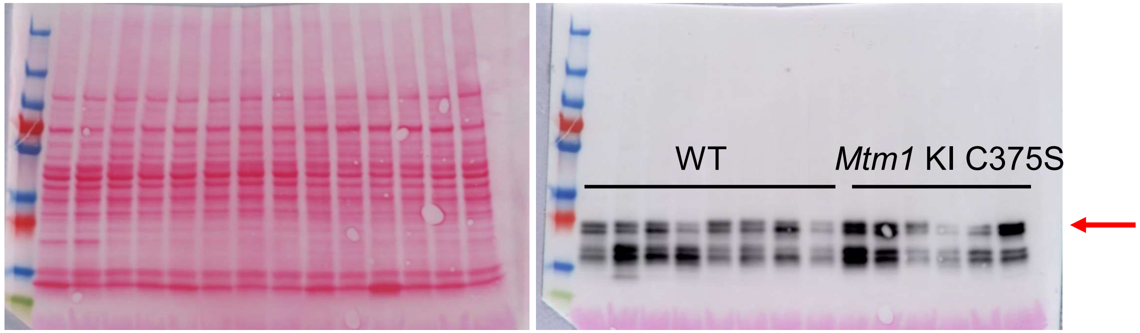

Primary antibody: anti-4EBP1 (rabbit, 1:1000, Cell Signaling Technology, #9644),

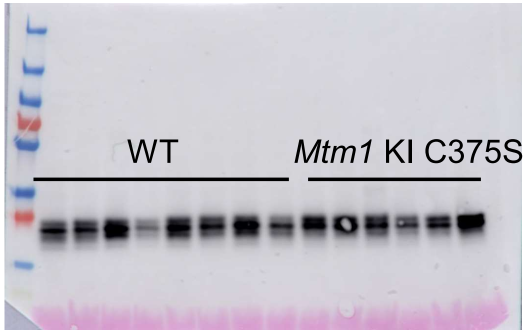

Full unedited blot for figure 7D

Primary antibody: anti-LC3 (rabbit, 1:1000, Cell Signaling Technology, #9206)

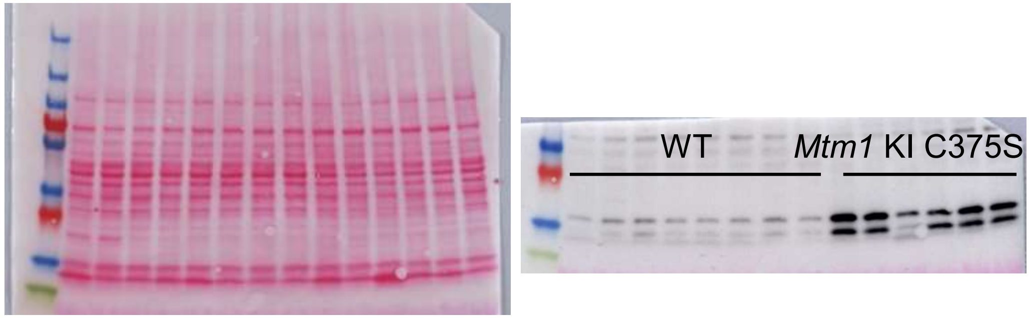

Full unedited blot for figure 7E

Primary antibody: anti-p62 (mouse, 1:1000, Novus Biologicals, #H00008878-M01)

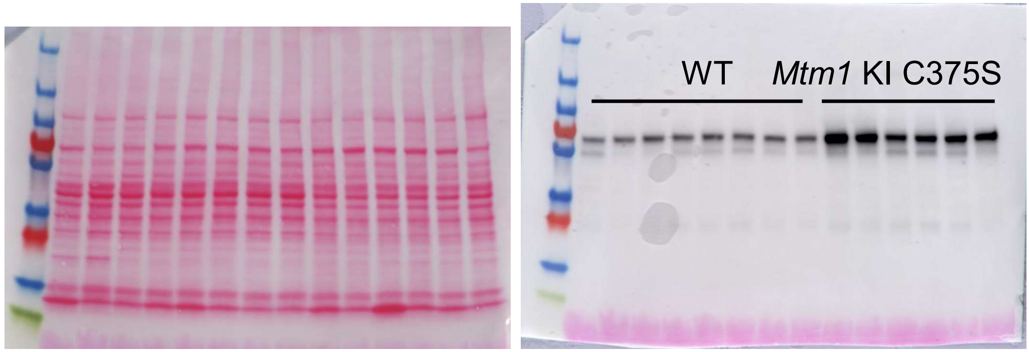

Full unedited blot for supplementary Figure 1A

Primary antibody: anti-MTM1 (rabbit, 1:700, homemade targeting the C-terminal end, #2827)

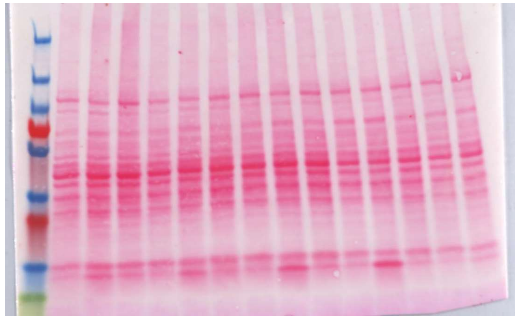

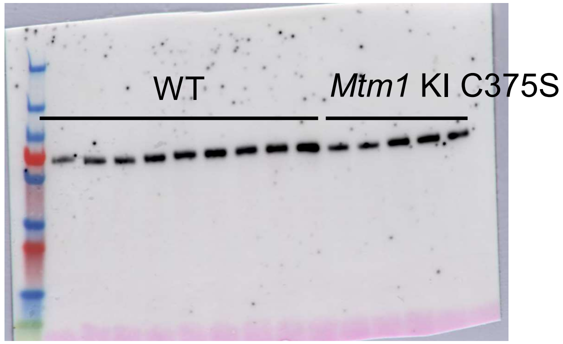

Full unedited blot for supplementary Figure 1B

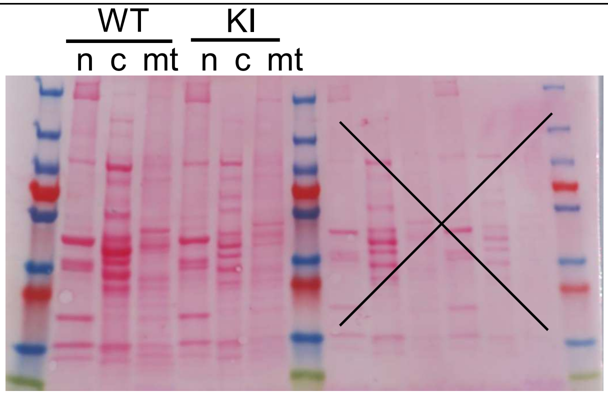

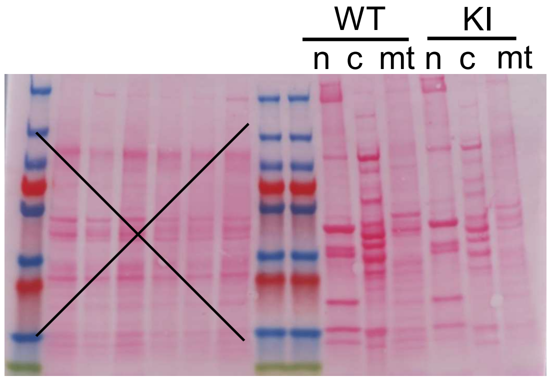

Primary antibody: anti-TOMM20 (rabbit, 1:1000, abcam, #ab78547)

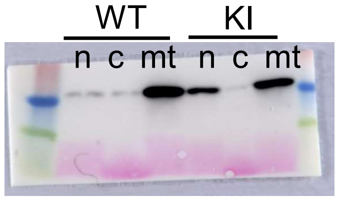

Primary antibody: anti-MTM1 (rabbit, 1:700, homemade targeting the C-terminal end, #2827)

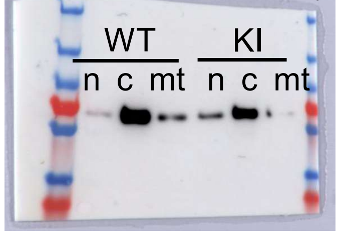

Primary antibody: anti-Lamin A (rabbit, 1:800, abcam, ab26300)

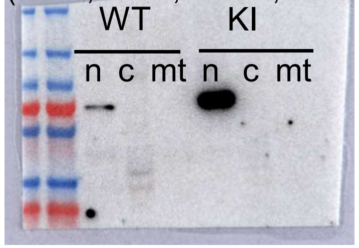

Primary antibody: anti-GAPDH (mouse, 1:1000, MERCK, #MAB374)

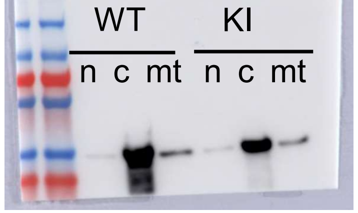

Full unedited blot for supplementary Figure 5F

Primary antibody: anti-DNM2 (rabbit 1:1000, homemade targeting the proline-rich domain, 2865)

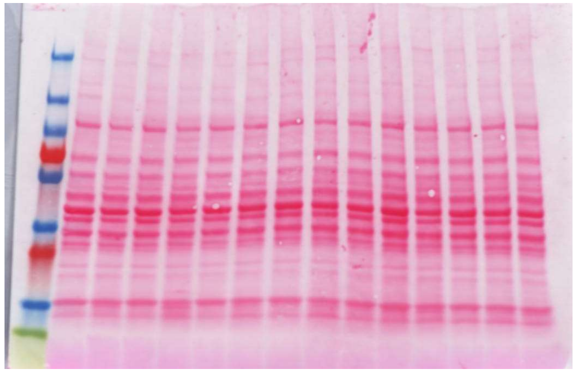

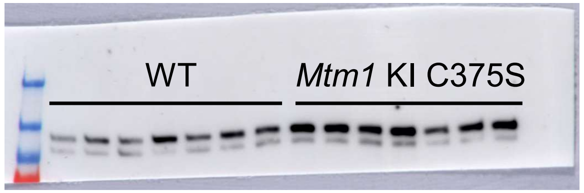

Full unedited blot for supplementary Figure 5G

Primary antibody: anti-BIN1 (rabbit, 1:1000, R3623; homemade targeting the SH3 domain, #3623)

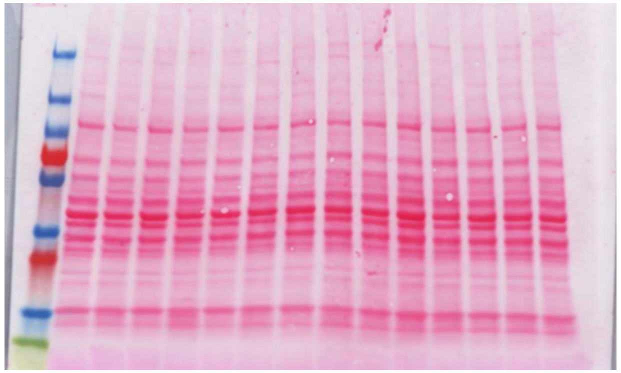

WT *Mtm1* KI C375S

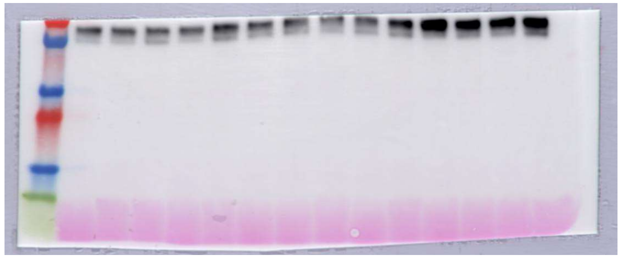

Full unedited blot for supplementary Figure 7A

Primary antibody: anti-prohibitin (rabbit 1:1000, abcam, #ab28172)

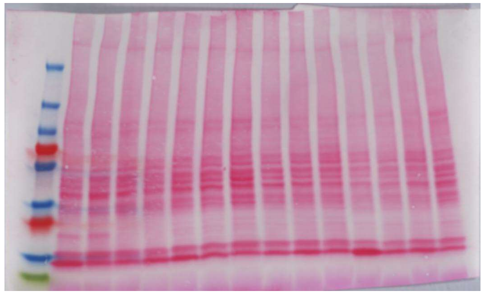

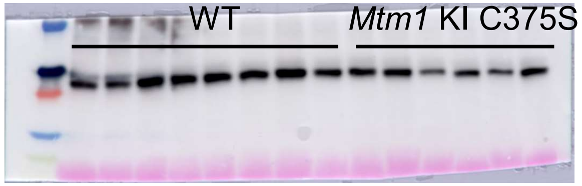

Supplement: Unedited blot and gel images [file jciinsight-10-189286-s148.pdf]
